# Human endogenous retrovirus K contributes to a stem cell niche in glioblastoma

**DOI:** 10.1172/JCI167929

**Published:** 2023-07-03

**Authors:** Ashish H. Shah, Sarah R. Rivas, Tara T. Doucet-O’Hare, Vaidya Govindarajan, Catherine DeMarino, Tongguang Wang, Leonel Ampie, Yong Zhang, Yeshavanth Kumar Banasavadi-Siddegowda, Stuart Walbridge, Dragan Maric, Marta Garcia-Montojo, Robert K. Suter, Myoung-Hwa Lee, Kareem A. Zaghloul, Joseph Steiner, Abdel G. Elkahloun, Jay Chandar, Deepa Seetharam, Jelisah Desgraves, Wenxue Li, Kory Johnson, Michael E. Ivan, Ricardo J. Komotar, Mark R. Gilbert, John D. Heiss, Avindra Nath

**Affiliations:** 1 University of Miami School of Medicine, Department of Neurosurgery, Miami, Florida, USA.; 2 National Institute of Neurological Disorders and Stroke, NIH, Bethesda, Maryland, USA.; 3 Neuro-Oncology Branch, Center for Cancer Research, National Cancer Institute, NIH, Bethesda, Maryland, USA.; 4 Georgetown University, Bioinformatics Section, Washington, DC, USA.; 5 Cancer Genetics Branch, National Human Genome Research Institute, NIH, Bethesda, Maryland, USA.

**Keywords:** Stem cells, Virology, Brain cancer, Neuronal stem cells

## Abstract

Human endogenous retroviruses (HERVs) are ancestral viral relics that constitute nearly 8% of the human genome. Although normally silenced, the most recently integrated provirus HERV-K (HML-2) can be reactivated in certain cancers. Here, we report pathological expression of HML-2 in malignant gliomas in both cerebrospinal fluid and tumor tissue that was associated with a cancer stem cell phenotype and poor outcomes. Using single-cell RNA-Seq, we identified glioblastoma cellular populations with elevated HML-2 transcripts in neural progenitor–like cells (NPC-like) that drive cellular plasticity. Using CRISPR interference, we demonstrate that HML-2 critically maintained glioblastoma stemness and tumorigenesis in both glioblastoma neurospheres and intracranial orthotopic murine models. Additionally, we demonstrate that HML-2 critically regulated embryonic stem cell programs in NPC-derived astroglia and altered their 3D cellular morphology by activating the nuclear transcription factor OCT4, which binds to an HML-2–specific long-terminal repeat (LTR5Hs). Moreover, we discovered that some glioblastoma cells formed immature retroviral virions, and inhibiting HML-2 expression with antiretroviral drugs reduced reverse transcriptase activity in the extracellular compartment, tumor viability, and pluripotency. Our results suggest that HML-2 fundamentally contributes to the glioblastoma stem cell niche. Because persistence of glioblastoma stem cells is considered responsible for treatment resistance and recurrence, HML-2 may serve as a unique therapeutic target.

## Introduction

Making up approximately 7%–8% of the human genome, human endogenous retroviruses (HERVs) are genomic remnants of exogenous retroviral germline infections (1–3). Because their function was largely elusive, HERVs were previously considered “junk DNA,” but now there is evidence that certain HERVs have retained evolutionary function that benefits their hosts, e.g., HERV-W (syncytin-1) plays a role in placental development (4, 5). The most recently integrated retrovirus, HERV-K (HML-2), is the most active HERV, and it possesses a nearly intact proviral genome and ORFs for major viral proteins gag, pol, pro, and env (1, 6, 7). In most normal cells, HML-2 transcription is silenced by a variety of epigenetic mechanisms, including histone and DNA methylation and chromatin remodeling (8). However, in certain cancers with marked epigenetic dysregulation, overexpression of HML-2 transcripts and retroviral proteins has been noted in both serum and tumor tissues (1, 9–13). Several studies have suggested that HML-2 may play a role in oncogenesis through several mechanisms, including cellular membrane fusion, LTR-mediated activation of oncogenic promoters, inactivation of tumor suppressors, and direct protein expression (1, 14, 15). Accordingly, HML-2 expression has been implicated in several oncogenic processes, including cellular proliferation, stem cell pluripotency, and cellular migration (11, 16–20). However, for malignant gliomas specifically, the presence and function of HML-2 has remained elusive (21). In this report, we conducted a comprehensive translational investigation of HML-2 expression in glioblastoma (GBM) and its role in maintaining the cancer stem cell phenotype.

## Results

### HML-2 is overexpressed in GBM.

**Figure 1:**
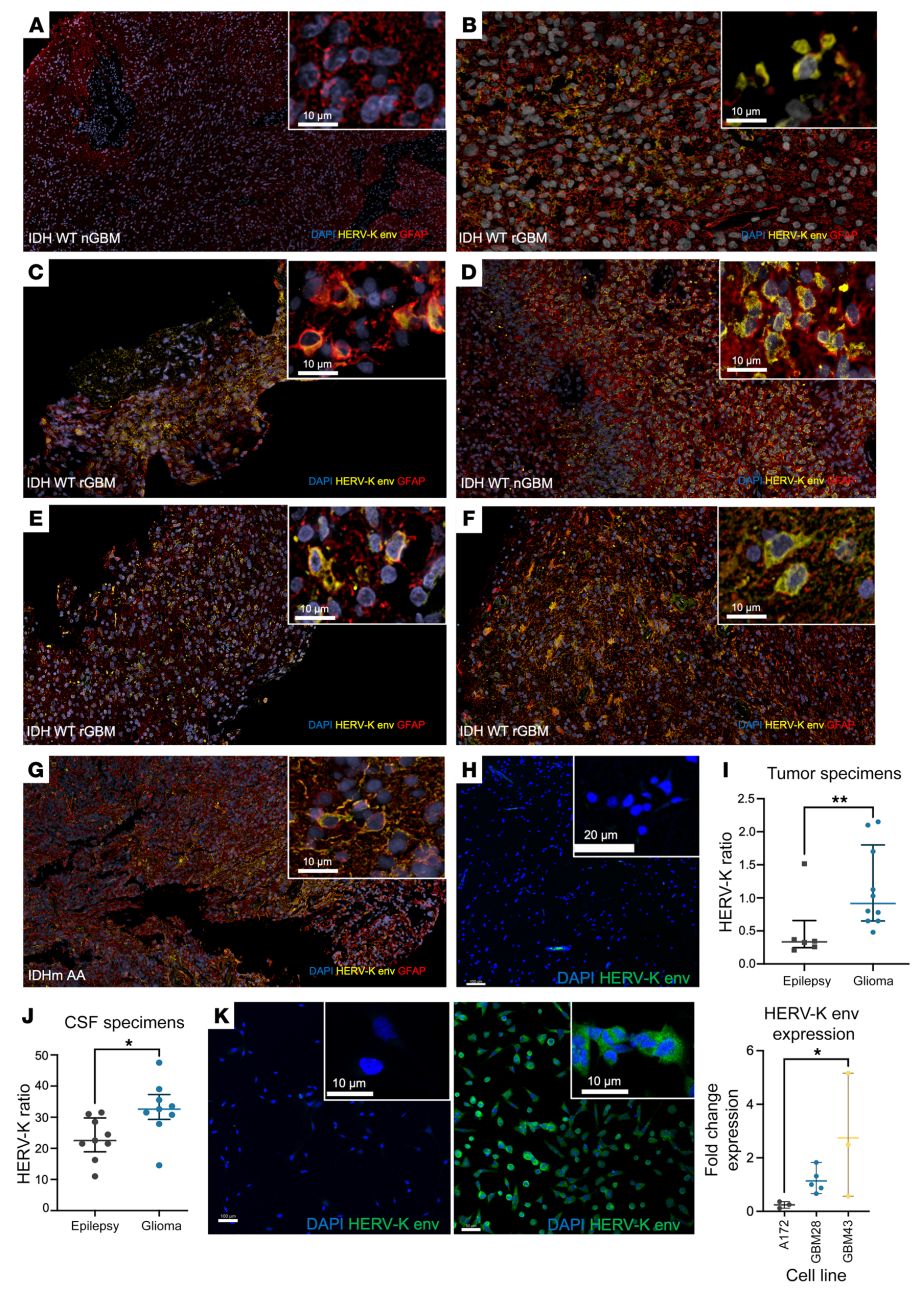
HML-2 envelope protein is elevated in glioblastoma. (**A**–**G**) Envelope protein is heterogeneously expressed in glioblastoma tissue using immunofluorescence and is expressed in most patients with glioblastoma (*n* = 8; 89%) (**H**) HERV-K envelope protein is not expressed in epilepsy control tissue (temporal neocortex). (**I**) HERV-K envelope RNA and *HPRT* copy numbers from epilepsy tissue and glioma samples were measured by digital-droplet PCR. HERV-K RNA/*HPRT* ratio and absolute copy numbers were elevated in patients with glioma compared with patients with epilepsy in the control group (Mann-Whitney test, **P = 0.01; mean = 1.15 ± 0.2 vs. 0.5 ± 0.2). (**J**) HERV-K DNA and *RPP30* copy numbers from perilesional CSF were measured by digital-droplet PCR. The absolute value of HERV-K DNA/*RPP30* of patients with high-grade gliomas (mean = 35.2 ± 8.8; *n* = 9) was significantly greater than that of patients with epilepsy (unpaired *t* test, *P = 0.02; 23.1 ± 6.7, *n* = 9). (**K**) HERV-K envelope protein is expressed in patient-derived glioma neurospheres (middle) but not widely expressed in established A172 adherent glioma cell lines (left). HERV-K *ENV* RNA transcripts as measured by qPCR correlate to protein expression: patient-derived neurosphere cell line GBM43 had significantly higher HERV-K *ENV* RNA than A172 (ANOVA, *P < 0.05) (right). Original magnification, ×10 (**A**–**G**). Scale bars: 10 μm (**A**–**G** and **K**, insets, and **K**); 20 μm (**H**, inset); 50 μm (**K**, right); 100 μm (**H** and **K**, left).

To evaluate the expression of HML-2 in GBM, we performed multiplex immunofluorescence, RNA in situ hybridization, and digital-droplet PCR (ddPCR) on samples from patients with glioma and individuals with epilepsy (Supplemental Table 1; supplemental material available online with this article; https://doi.org/10.1172/JCI167929DS1). Using multiplex immunofluorescence, most GBM samples stained positive for HERV-K env protein, whereas we observed no expression in cortical epilepsy samples (Figure 1, A–H). Using ddPCR, we then validated overexpression of HML-2 RNA in malignant glioma samples compared with nontumoral epilepsy tissues (HML-2/HPRT1 ratio, Figure 1I). Next, we investigated whether HML-2 can be isolated from cerebrospinal fluid (CSF) from patients with glioma and nontumoral epilepsy. Using a previously validated ratio that controls for genomic DNA, the HML-2 ratio was significantly higher in the CSF of patients with glioma compared with that of patients with epilepsy (HML-2/RPP30, Figure 1J). Patient-derived GBM neurosphere lines GBM28 and GBM43 demonstrated increased expression of HERV-K env protein compared with the established non-stem-like glioma cell line, A172, using immunofluorescence and quantitative PCR (qPCR) (Figure 1K). Additionally, Western blotting validated significantly increased expression of HML-2 env protein in GBM tumors compared with that in matched normal brain samples (Supplemental Figure 1).

**Figure 2:**
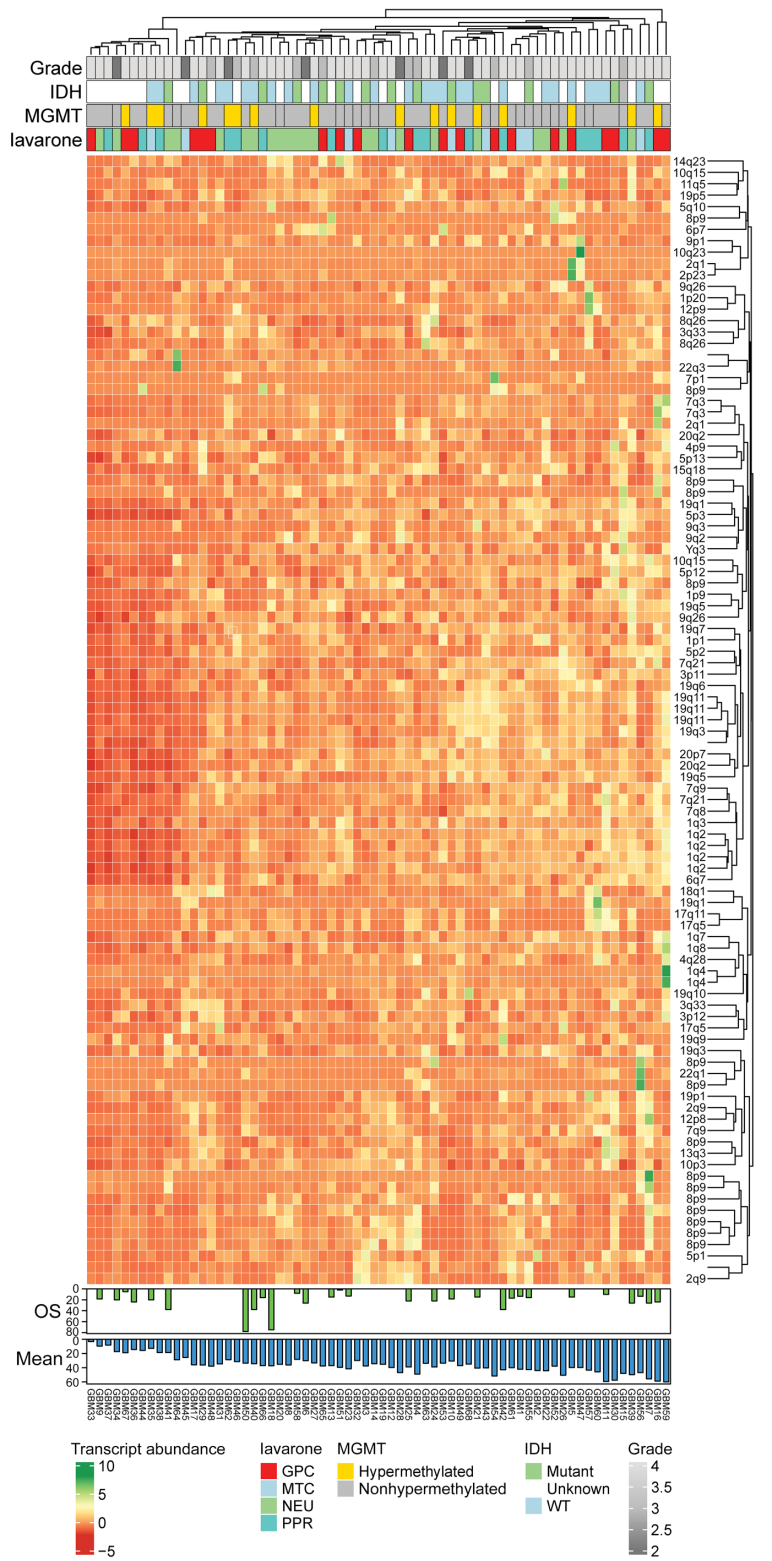
HERV-K is transcriptionally activated in samples from patients with high-grade glioma. Summary of bulk RNA-Seq data for 71 patients with high-grade glioma across 123 HML-2 loci, alongside IDH mutation status, MGMT hypermethylation status, tumor grade, Verhaak classification, mean HML-2 expression, and overall survival (OS).

**Figure 3:**
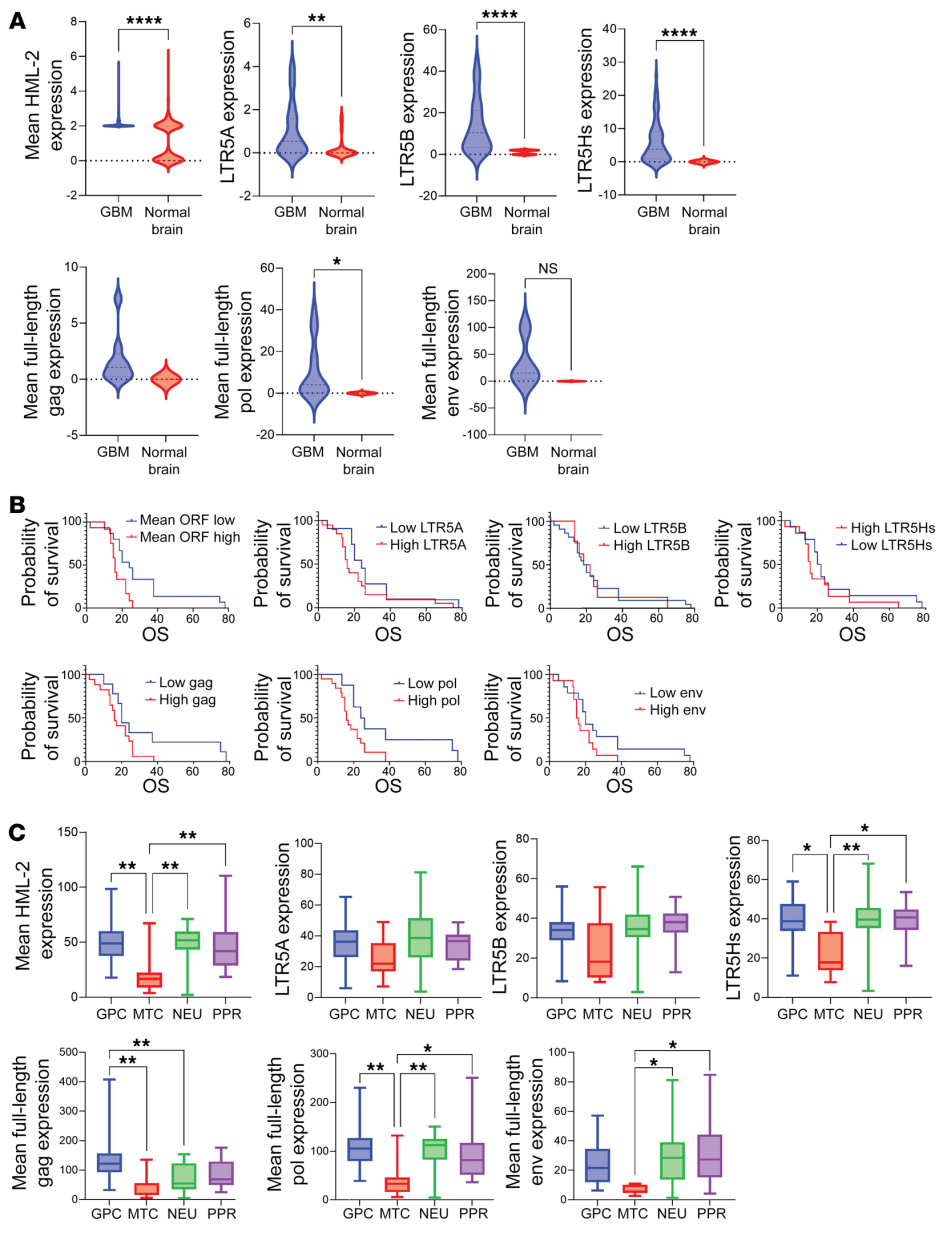
HERV-K corresponds to a poor overall outcome. (**A**) Mean HML-2 expression was significantly higher in high-grade glioma tissue (*n* = 71) relative to that of normal control brain tissue (*t* test, *P* < 0.0001; 2.17 ± 0.02 vs. 1.08 ± 0.06; *n* = 100). Mean *LTR5A*, *LTR5B*, and *LTR5Hs* expression was significantly higher in GBM tissue relative to that in normal control tissue (*t* test, **P = 0.0018, ****P < 0.0001). Mean full-length HML-2 polymerase expression was overexpressed compared with that in normal control brain tissue (*t* test, *P = 0.01). (**B**) Impact of transcript expression on OS in patients with GBM. Patients with GBM with higher expression of HML-2 with intact ORF demonstrated significantly worse OS relative to patients with lower mean HML-2 expression (Mantel-Cox test, *P* = 0.011). Patients with GBM with higher expression of HML-2 full-length *gag* (Mantel-Cox test, *P* = 0.027), *pol* (Mantel-Cox test, *P* = 0.0068) and *env* (Mantel-Cox test, *P* = 0.028) demonstrated worse OS relative to patients with lower respective HML-2 transcripts. No significant differences in OS between patients with high and low *LTR5A*, *LTR5B*, and *LTR5Hs* expression were found. Cutoff values for high and low expressing groups were determined using maximally ranked statistics as detailed in the R package survminer. The cutoffs (FPKM) used were as follows: mean ORF, 88.3; LTR5A, 30.73; LTR5B, 39.46; LTR5Hs, 39.02; gag2, 8.20; pol2, 15.54; env2,23.50. (**C**) Relationship between HML-2 coding loci and Verhaak glioma classification. There were no significant differences in *LTR5A* or *LTR5B* expression across Verhaak states. However, MTC tumors demonstrated significantly reduced expression of HML-2 loci with *LTR5Hs* relative to GPC, PPR, and NEU tumors (ANOVA, *P* = 0.01, 0.02, 0.002). With respect to mean full-length gag expression, GPC lineage tumors had higher expression relative to that of MTC and NEU lineage tumors (ANOVA, *P* = 0.001, 0.005). MTC lineage tumors demonstrated reduced mean full-length pol expression relative to that of GPC, NEU, and PPR lineage tumors (ANOVA, multiple testing corrections, *P* = 0.003, 0.008, 0.01). MTC lineage tumors also demonstrated reduced mean full-length env expression with respect to NEU and PPR lineage tumors (*P* = 0.02, 0.01). GPC, glycolytic/plurimetabolic cellular state; MTC, mitochondrial; NEU, neuronal; PRR, proliferative/progenitor. *P < 0.05, **P < 0.01.

We utilized a custom bioinformatic pipeline (TE-Transcripts) to quantify loci-specific expression of HML-2 (123 unique loci) in human gliomas and normal brain in 71 glioma samples (Figure 2). Using this data set, we identified overexpression of several HML-2 ranscripts (an HML-2–specific long-terminal repeat [*LTR5Hs*], *ENV*, *POL*, *GAG*) compared with age- and sex-matched normal brain controls (*n* = 100) (Figure 3A). Using the new GBM classification system, the mitochondrial subtype had significantly lower expression of HML-2 compared with the glycolytic, proliferative/progenitor, and neural subtypes (22). Due to accumulation of deletions and insertions during evolution, each locus for HML-2 contains divergent expression of retroviral coding genes *GAG*, *POL*, *ENV*, *LTR5A*, *LTR5B*, and *LTR5Hs*. Summary expression of loci containing full-length retroviral gene products revealed that high overall mean HML-2 correlated with a worse outcome for patients with high-grade glioma, which was maintained for individual gene products, *GAG*, *POL*, and *ENV* (Figure 3, B and C).

### Single-cell lineage tracing identifies an enriched HML-2 stem cell population.

**Figure 4:**
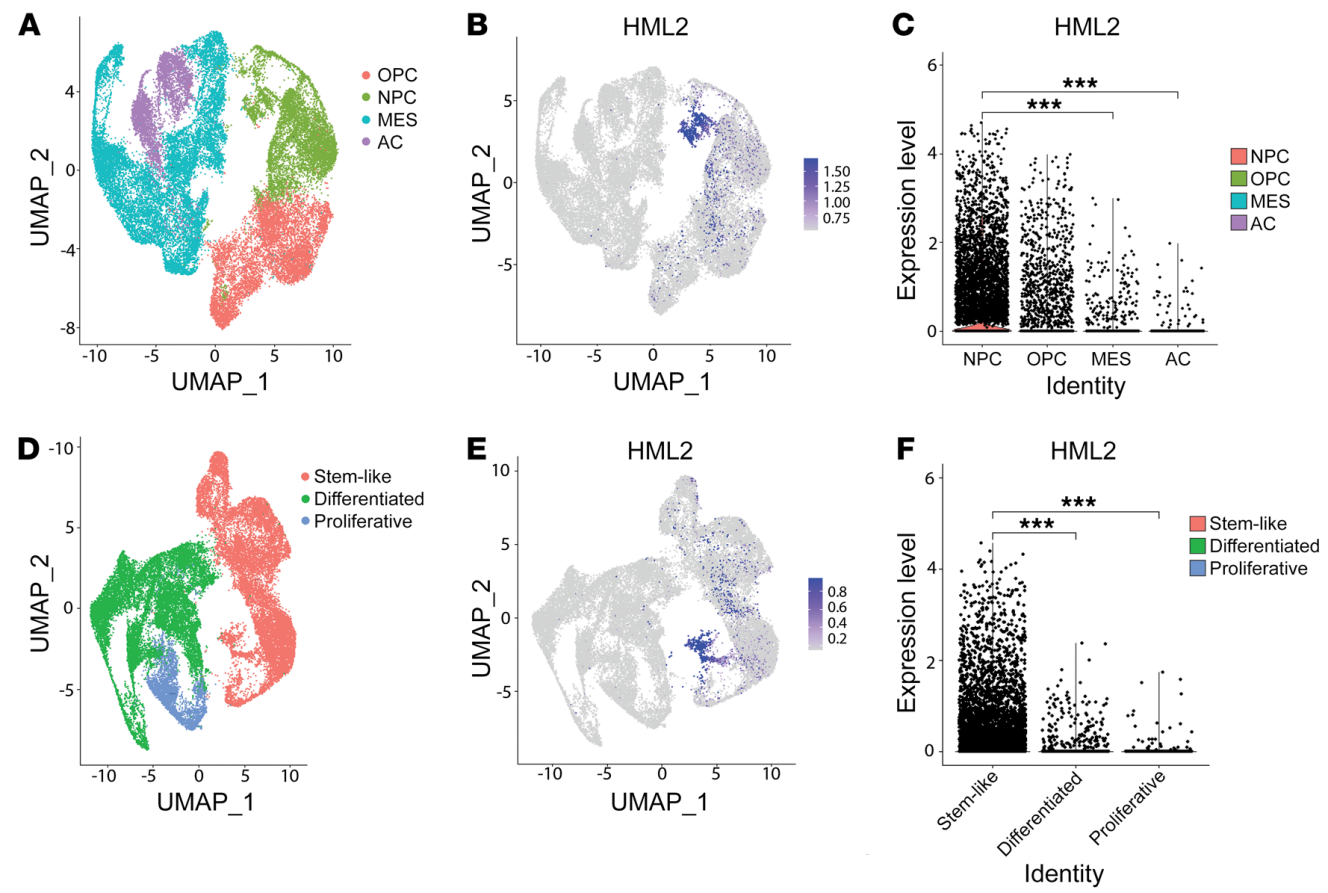
HML-2 is transcriptionally activated in neural stem–like glioma cells. (**A**) Mean HML-2 expression strongly colocalized to populations corresponding with an NPC cellular program. (**B**) Unsupervised cluster analysis all HML-2 loci distinguish subpopulations of GBM cellular states with dedifferentiated phenotype. (**C**) Mean HML-2 expression was significantly higher in NPC cells relative to mesenchymal (MES) and astrocyte (AC) cells (***P < 0.001). (**D** and **E**) HML-2 is spatially enriched in Stem cell-like populations relative to differentiated or proliferative populations. (**F**) Mean HML-2 expression was significantly higher among stem-like cells relative to differentiated and proliferative cells (***P < 0.001).

**Figure 5:**
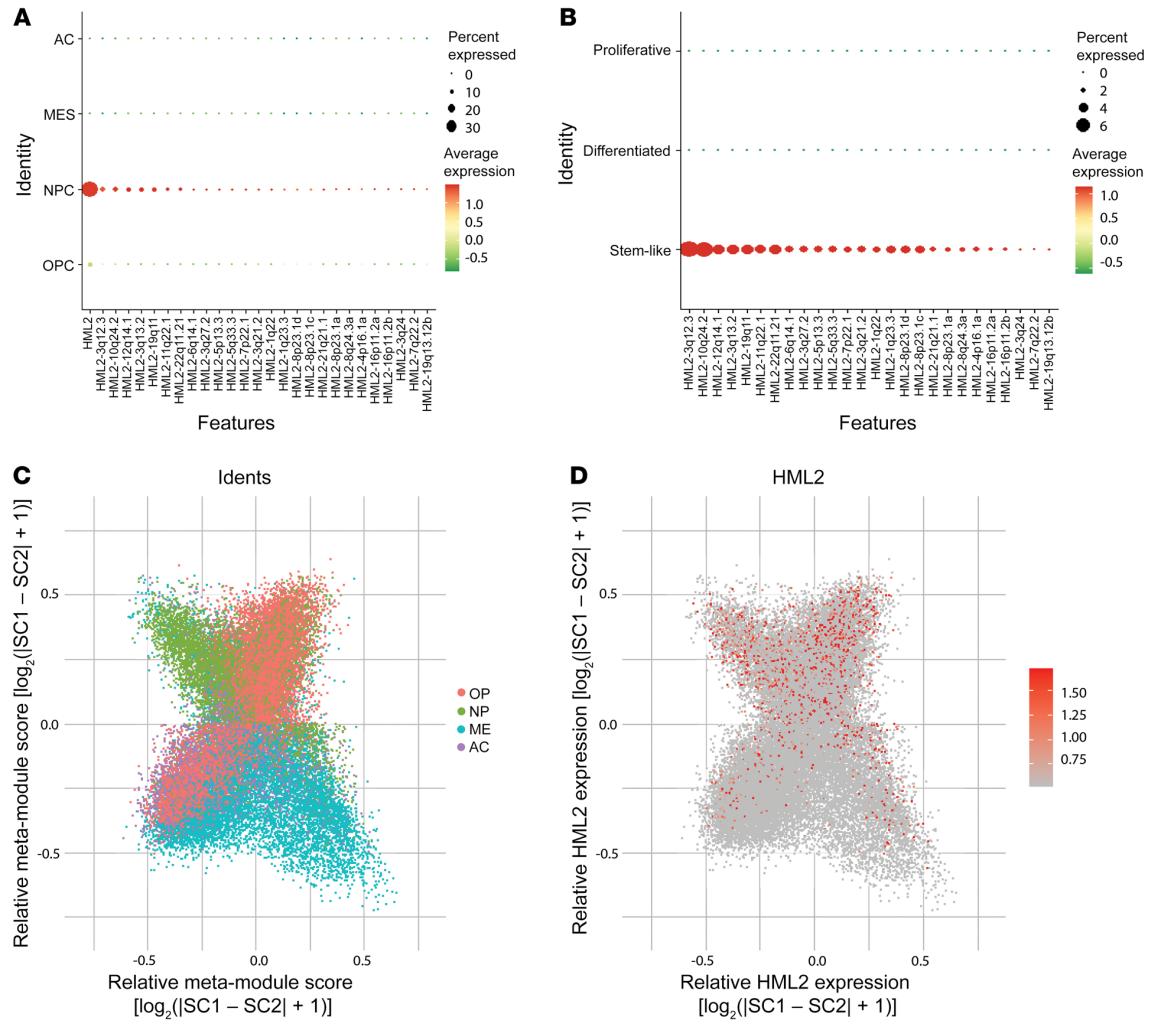
HML-2 is enriched in NPC and stem cell–like populations. (**A** and **B**) Twenty-six unique HML-2 loci were identified that were uniquely enriched in NPC and stem cell–like populations. (**C** and **D**) Distribution of cells by Neftel state meta-module scores reveals HML-2 enrichment in both oligodendrocyte-precursor cell–like (OPC-like) and NPC-like clusters.

To improve our understanding of locus-specific HML-2 expression in GBM cells, we conducted an unbiased transcriptomic assessment of single-cell RNA-Seq (scRNA-Seq) data from patient-derived tumor specimens. We first characterized HML-2 expression in 870 GBM cells isolated from 6 tumors, which was then validated in a larger data set that included 55,284 cells from 11 tumors (23, 24). To determine the transcriptomic relevance of HML-2 expression in single-cell clusters, we first stratified our data for only loci containing intact ORFs for HML-2 retroviral proteins (GAG, POL, ENV) and then correlated HML-2 expression to predefined malignant cellular programs (25). Cellular transcriptional states were defined previously as oligodendrocyte-precursor cell like (high PDGFR), astrocyte like (EGFR amplification), mesenchymal (NF1 alteration), and neural progenitor cell like (NPC, high CDK4) (25). In both independent data sets, the single-cell data revealed a cluster of cellular subpopulations of GBM with differentially expressed high HML-2 expression in NPC-like and stem-like cell clusters (24) (Figure 4 and Figure 5 and Supplemental Figure 2). Additionally, the HML-2 clusters comprised subpopulations of several parent tumors, suggesting that HML-2 expression was independent of the tumor origin, as shown in Supplemental Figure 3 (25). Our analysis demonstrated that HML-2 expression was specific to cellular subpopulations with enriched NPC-like signatures and that this HML-2 expression was significantly higher when compared with astrocyte-like and mesenchymal cells, as shown in Supplemental Tables 2 and 3. Because the NPC-like clusters have been suggested to play a role in glioma stemness, we sought to determine the spatial relation of differentially expressed HML-2 loci to these stem cell–like populations (26, 27). After multiple-testing correction, our unbiased analysis identified several HML-2 loci that demonstrated substantial enrichment within the NPC-like subpopulation compared with other cellular states, as shown in Supplemental Figure 3, C and D. Quality control and preliminary principal component analysis data are shown in Supplemental Figure 4.

The *LTR5Hs* from HML-2 has been suggested to retain pervasive functionality as a transcription factor binding site and genomic enhancer for hundreds of downstream genes (28). LTRs are transposable elements that flank the internal coding region of HERVs and can act as mediators of retrotransposition or as promoter-binding sites for HERV transcription. The *LTR5Hs* from HERV-K has been suggested to play an essential role in maintaining stemness in early embryogenesis because it contains an active binding site for the transcription factor OCT4 (29, 30). Using the curated retrotransposable element database, HervD, we identified 8 loci with preserved *LTR5Hs* and 5 loci containing a near-full-length provirus with all coding genes (3q21.2, 7p22.1, 11q22.1, 8p23.1, 19q11, Hg38) that were overexpressed in stem-like cells compared with differentiated cells (31). Similarly, we analyzed summed HML-2 expression across all known loci among our scRNA-Seq samples. Our analysis demonstrated significantly higher total HML-2 expression in NPC lineage cells relative to all other investigated cell types, as shown in Supplemental Table 4 and in Supplemental Figure 3, E and F. Relative expression of HML-2 with respect to cluster identity is shown in Supplemental Figure 5. A summary of each differentially expressed HML-2 loci and their coding regions as curated by HervD can be found in Supplemental Table 5 (31). These HML-2 insertional sites (Hg38) neighbor several functional genes that play integral roles in cancer cell proliferation, growth, and the antiviral immune response, as shown in Supplemental Table 6. Overall, these results suggested that HML-2 transcripts play an important role in defining malignant stem cell states in GBM.

### HML-2 expression corresponds to a unique stem cell niche in malignant gliomas.

**Figure 6:**
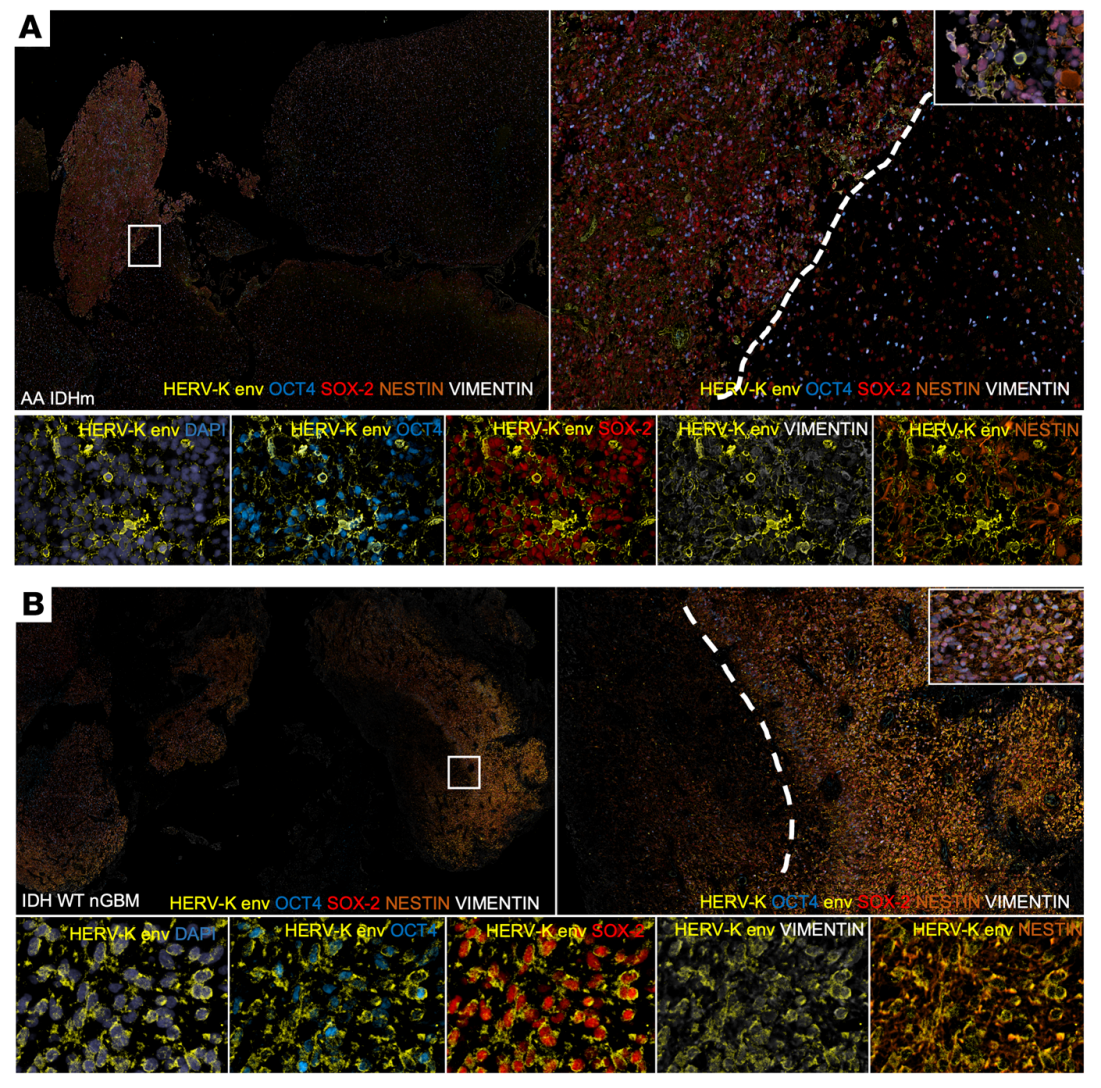
HML-2 envelope protein expression correlates to stem cell–enriched areas in glioblastoma. Using 11-plex whole-slide immunofluorescence, glioma stem cell markers and HERV-K localized to stem cell–rich areas containing abundant OCT4, Sox2, Nestin, and Vimentin expression in (**A**) IDHm anaplastic astrocytoma and (**B**) IDH WT GBM. Note that the histological section presented in **B** is from the same patient as shown in Figure 1D. Original magnification, ×4 (top left); ×10 (top right); ×20 (bottom rows).

Previously, we demonstrated that HERV-K was critical to maintaining stemness and pluripotency through the nuclear transcription factor OCT4 (32). Because the glioma stem cell niche is associated with overexpression of several proteins, including OCT4, Nestin, Vimentin, and Sox-2, we sought to visualize HML-2 expression in situ with multiplex immunofluorescence. We found HML-2 expression in the same cells and tissues that expressed stem cell markers, namely OCT4, Nestin, Vimentin, and Sox2 in patients with high-grade glioma (Figure 6 and Supplemental Figure 6A). Correspondingly, multivoxel segmentation revealed a high-correlation of expression of stem cell markers and HML-2 in stem cell–rich and –depleted areas (Supplemental Figure 6B).

### HML-2 is critical in maintaining the cancer stem cell phenotype in GBM.

**Figure 7:**
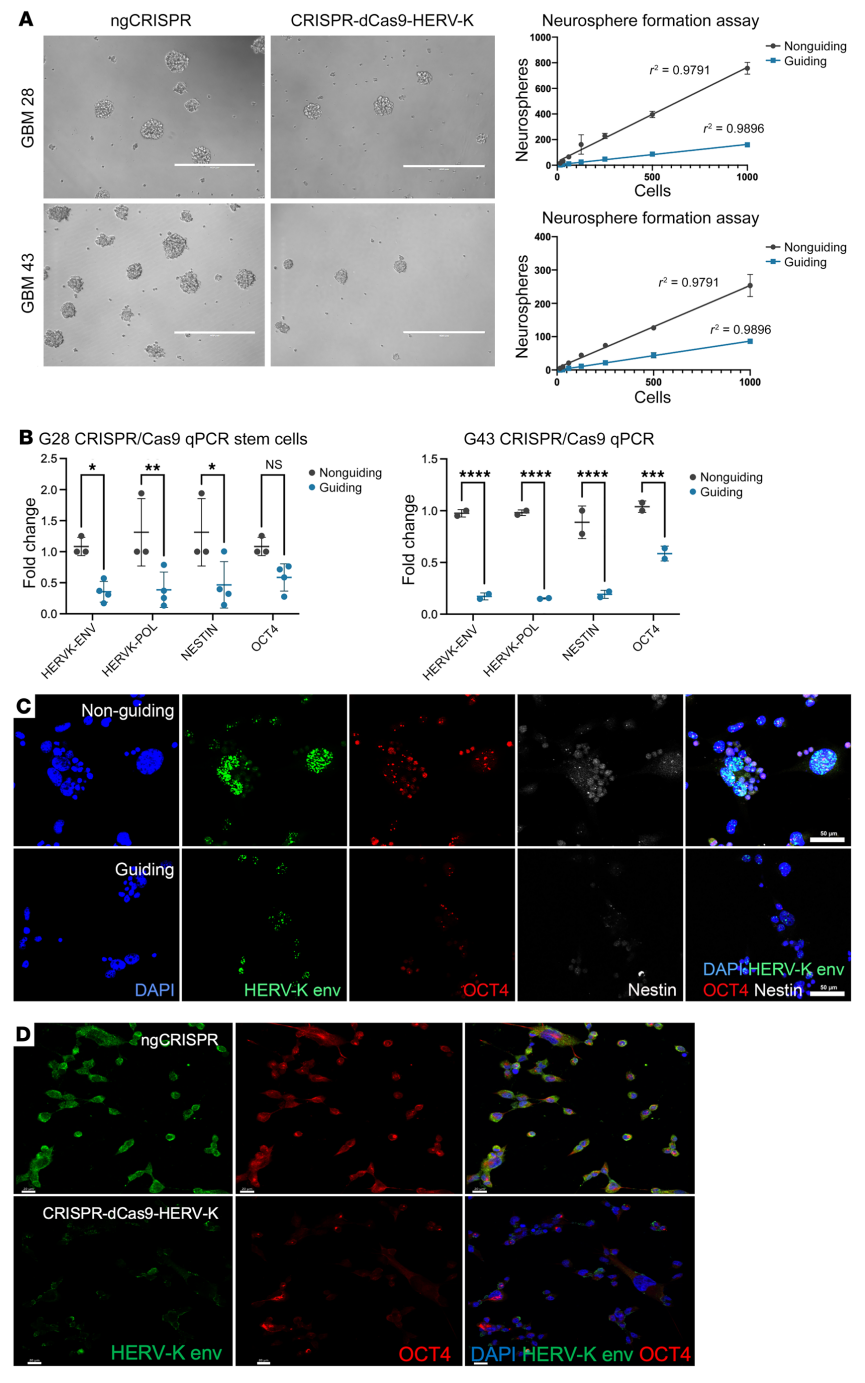
Downregulation of HERV-K using CRISPRi reduces the glioblastoma stem cell phenotype. (**A**) CRISPR/dCas9-HERV-K significantly reduced neurosphere formation compared with nonguiding CRISPR/dCas9 in 3 biological replicates (2-way ANOVA, *P* < 0.0001). Scale bars: 400 μm. (**B**) CRISPR/dCas9-HERV-K significantly reduced HERV-K *env*, *Polymerase*, and *Nestin* transcripts in GBM28 and GBM43. OCT4 transcripts were significantly reduced in GBM 43. (Unpaired *t* test, *P < 0.05, **P < 0.01, ***P < 0.001, ****P < 0.0001). (**C**) RNA in situ hybridization demonstrated reduced HERV-K env, OCT4, and Nestin transcripts 48 hours after transfection with CRISPR/dCas9-HERV-K plasmids. Scale bars: 50 μm. (**D**) Protein immunofluorescence demonstrated reduced HERV-K envelope and OCT4 expression 72 hours after transfection with CRISPR/dCas9-HERV-K plasmids compared with ngCRISPR/dCas9 in patient-derived glioma neurospheres. Scale bars: 20 μm.

We downregulated HML-2 expression in glioma cells using a CRISPR interference (CRISPRi) construct to further elucidate the relationship between HML-2 exæpression and glioma stemness (13). The CRISPRi HML-2 construct targets the consensus sequence of the HML-2 LTR with 4 guide RNAs using a CRISPR/dCas9 construct. Downregulation of HML-2 transcription resulted in a decrease in cellular adhesion and viability in atypical teratoid/rhabdoid cells; therefore, we conducted a neurosphere formation assay using our glioma cell lines (13). Neurosphere formation is an indirect indication of increased tumor aggressivity and diminished progression-free survival in gliomas and a hallmark of glioma stem cells (33–35). At 10 days after transfection, CRISPRi-HML-2–transfected GBM cells had significantly reduced neurosphere formation compared with the no-guide CRISPRi–transfected (ngCRISPRi-transfected) cells, supporting the role of HML-2 transcription in cellular proliferation (Figure 7A). We confirmed knockdown of HML-2 transcription with qRT-PCR and with RNA in situ hybridization on the transfected GBM lines (GBM28 and GBM43) (Figure 7B). *OCT4* transcripts were also reduced in CRISPRi-HML-2–transfected cells with RNA in situ hybridization 48 hours after transfection; furthermore, we detected decreased HML-2 env and OCT4 proteins by immunostaining at 72 hours after transfection in CRISPRi-HML-2 glioma neurospheres compared with the ngCRISPRi construct (Figure 7, C and D).

### HML-2 promotes an aggressive GBM phenotype.

**Figure 8:**
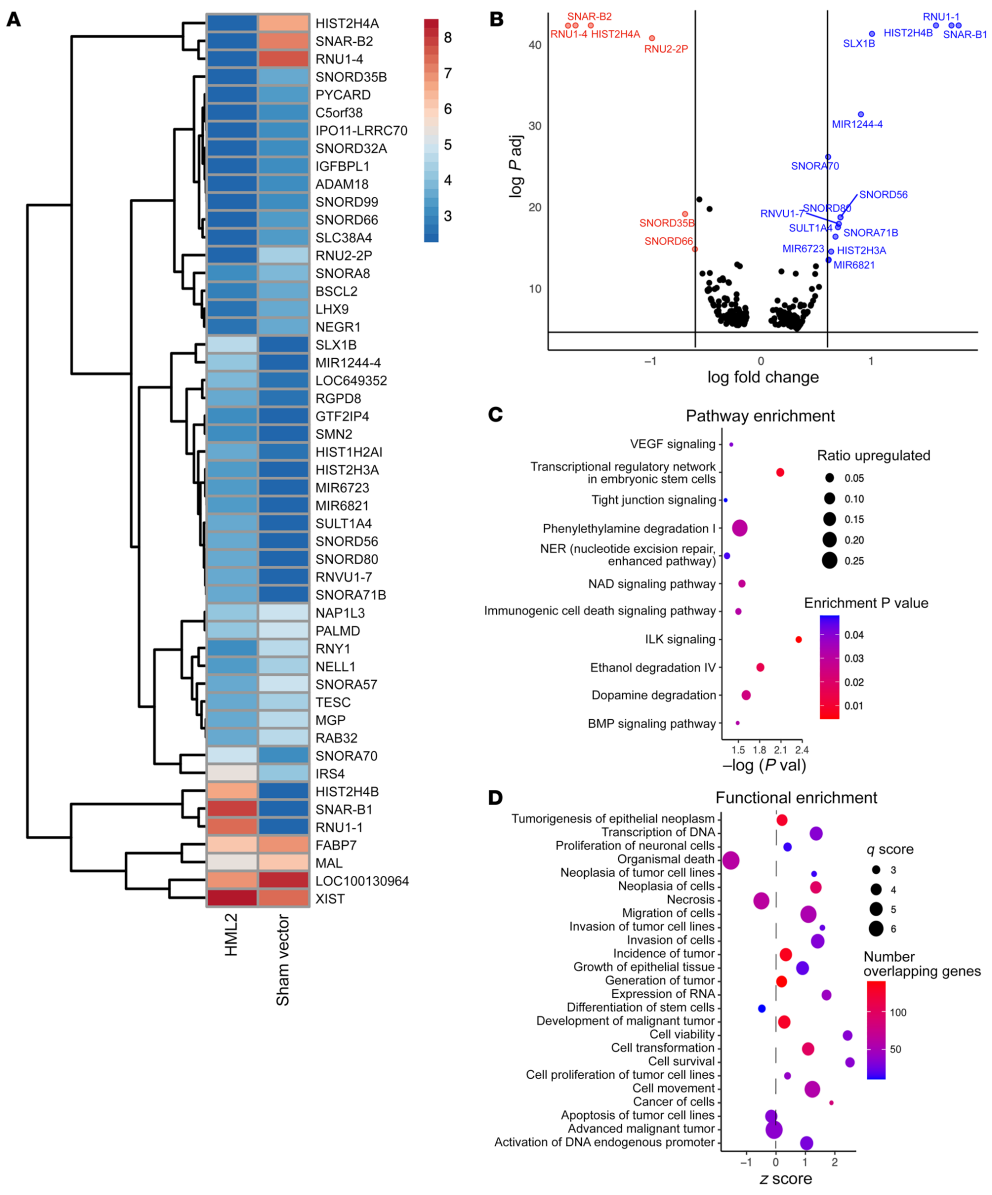
HML-2 transcriptionally activates embryonic stem cell programs in NSC-derived astrocytes. (**A**) Differential expression of genes in CD34^+^ astroglial cells transfected with HML2 consensus plasmid vs. sham vector. (**B**) Differential gene expression analysis demonstrated activation of stem cell regulatory genes in CD34^+^ astroglia transfected with consensus HML-2 plasmid compared with sham vector at 72 hours (*P* < 0.05, |logFC| > 0.58, multiple testing corrections). Numbers 3–8 are differential expression counts. (**C** and **D**) Transfection with HML2 consensus plasmid resulted in upregulation of embryonic stem cell pathways (integrin linked kinase [ILK] signaling, transcriptional regulatory network) (*P*_enrich_ < 0.05) and cellular functions (*P*_enrich_ < 0.05) that resulted in increased cellular proliferation, migration, invasion, and dedifferentiation in CD34^+^ astroglial cells.

**Figure 9:**
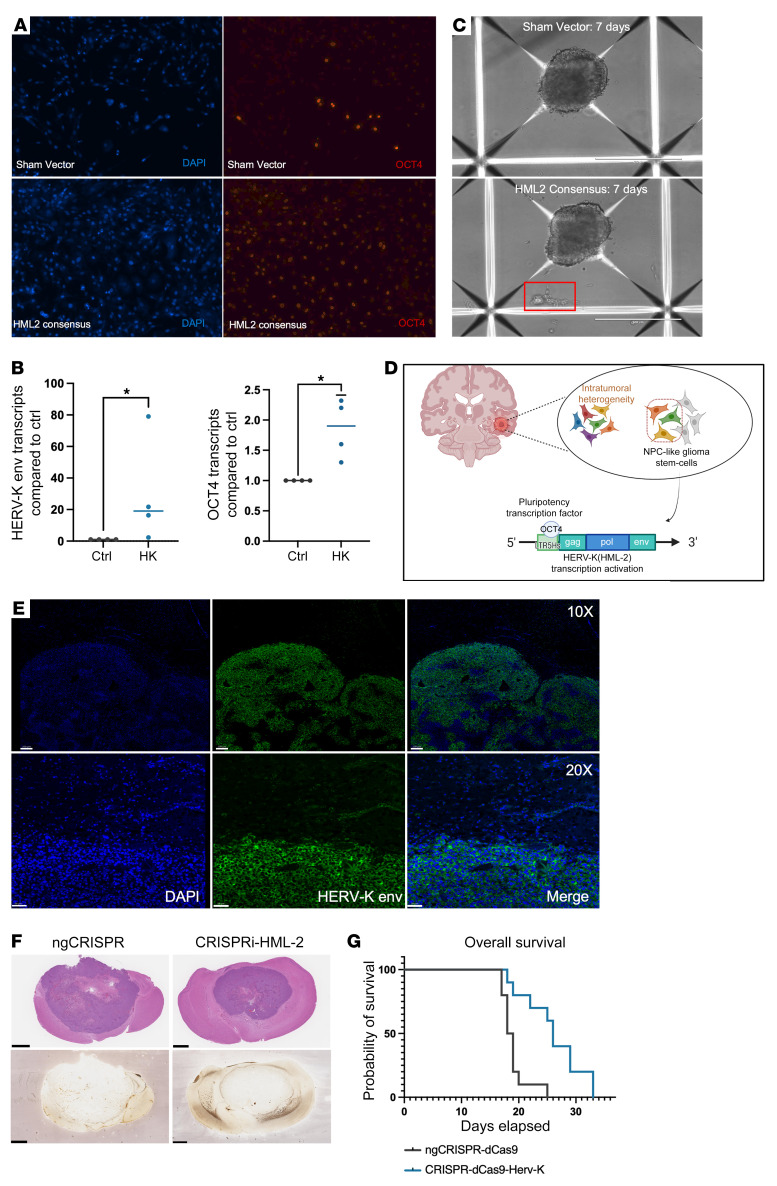
HERV-K induces astrocytic dedifferentiation and a tumorigenic phenotype. (**A**) Transfection of glioblastoma neurospheres with pC-HK results in increased HML-2 envelope protein expression and increased OCT4 expression (original magnification, ×20). (**B**) HML2 leads to increased expression of HERV-K ENV (*P* < 0.03) and OCT4 (*P* < 0.03) transcripts in CD34^+^ astroglia. *P < 0.05. (**C**) HML2 also induced increased cellular migration and satellitosis in NSC-derived astrocytes. Scale bars: 400 μm. (**D**) Schematic demonstrating OCT4-mediated transcriptional activation of HERV-K within integrated NPC-like cellular state. Image created with BioRender. (**E**) Untransfected cells are responsible for intracranial tumor engraftment and retain prominent HERV-K envelope expression. Images were taken (original magnification, ×10 and ×20) at the tumor-parenchymal interface. Normal mouse brain does not express HERV-K envelope protein. Scale bars: 200 μm. (**F**) Engrafted tumors share similar morphology among ngCRISPR (day 18) and CRISPR/dCas9-HERV-K (day 33), with characteristic central necrosis on H&E staining and silver staining. Scale bars: 1 mm. (**G**) Transient inhibition of HERV-K improved survival in patient-derived orthotopic xenograft glioma models. Mice implanted with CRISPR/dCas9-HERV-K demonstrated longer overall survival than ngCRISPR/dCas9 controls (Grehan-Breslow-Wilcoxon test, *P* = 0.0008; OS, 26 days vs. 18.6).

We hypothesized that stem cell–like features in the GBM cell lines could be a consequence of HML-2 expression. To investigate this possibility, we derived astrocytes from CD34^+^ neural stem cells and transfected them with an HML-2 consensus sequence plasmid in 3D culture (32). Using RNA-Seq, we were able to identify upregulation of several cellular programs involved in transcriptional regulation of embryonic stem cells and integrin linked kinase signaling. HML-2 significantly activated several cellular genes that contributed to cancer cell invasion, migration, proliferation, and dedifferentiation (Figure 8 and Supplemental Tables 7 and 8). Consistent with these findings, we found that HML-2 induced OCT4 expression in NSC-derived astrocytes using immunofluorescence and qPCR (Figure 9, A and B). HML-2 also induced significant morphological changes in cultured NSC-derived astrocytes that promoted cellular migration and satellitosis (Supplemental Video 1 and Figure 9C). Additionally, we found that HML-2 induced OCT4 expression in NSC-derived astrocytes using immunofluorescence and qPCR (Figure 9, A and B). The increased expression of OCT4 suggests a potentially bidirectional role between HML-2 and the nuclear transcription factor OCT4. Previously, the HML-2 *LTR5Hs* was demonstrated to contain a binding motif for the nuclear transcription factor OCT4 (30, 36, 37) (Figure 9D). Using a ChIP assay we found that OCT4 actively bound to the HML-2 LTR in our patient-derived GBM neurospheres and, thus, could potentially facilitate HML-2 transcription. There was no significant binding detected with our negative control in which antibody to OCT4 was replaced with a control IgG (Supplemental Figure 7G).

Having established the relationship between HML-2 and glioma stemness, we sought to test whether HML-2 affected the tumorigenicity of our patient-derived glioma neurosphere cell lines. We orthotopically implanted nucleofected GBM cells with either ngCRISPRi or CRISPRi HML-2 into the right frontal lobe of nude athymic (Foxn1nu) mice and monitored them for tumor growth. At the end of the observation period, all mice developed tumors at the site of injection, became moribund, and were sacrificed. Both tumors, ngCRISPRi and CRISPRi-HML2 knockdown, exhibited characteristic glial cell morphology with an invasive phenotype. Of note, HML-2 env expression remained highly expressed in engrafted tumors, suggesting that untransfected HML-2^+^ subpopulations drove tumorigenesis (Figure 9, E and F). The CRISPRi HML-2 knockdown mice survived significantly longer than mice injected with no-guide RNA (Figure 9G).

### HML-2 virions are formed in GBM.

**Figure 10:**
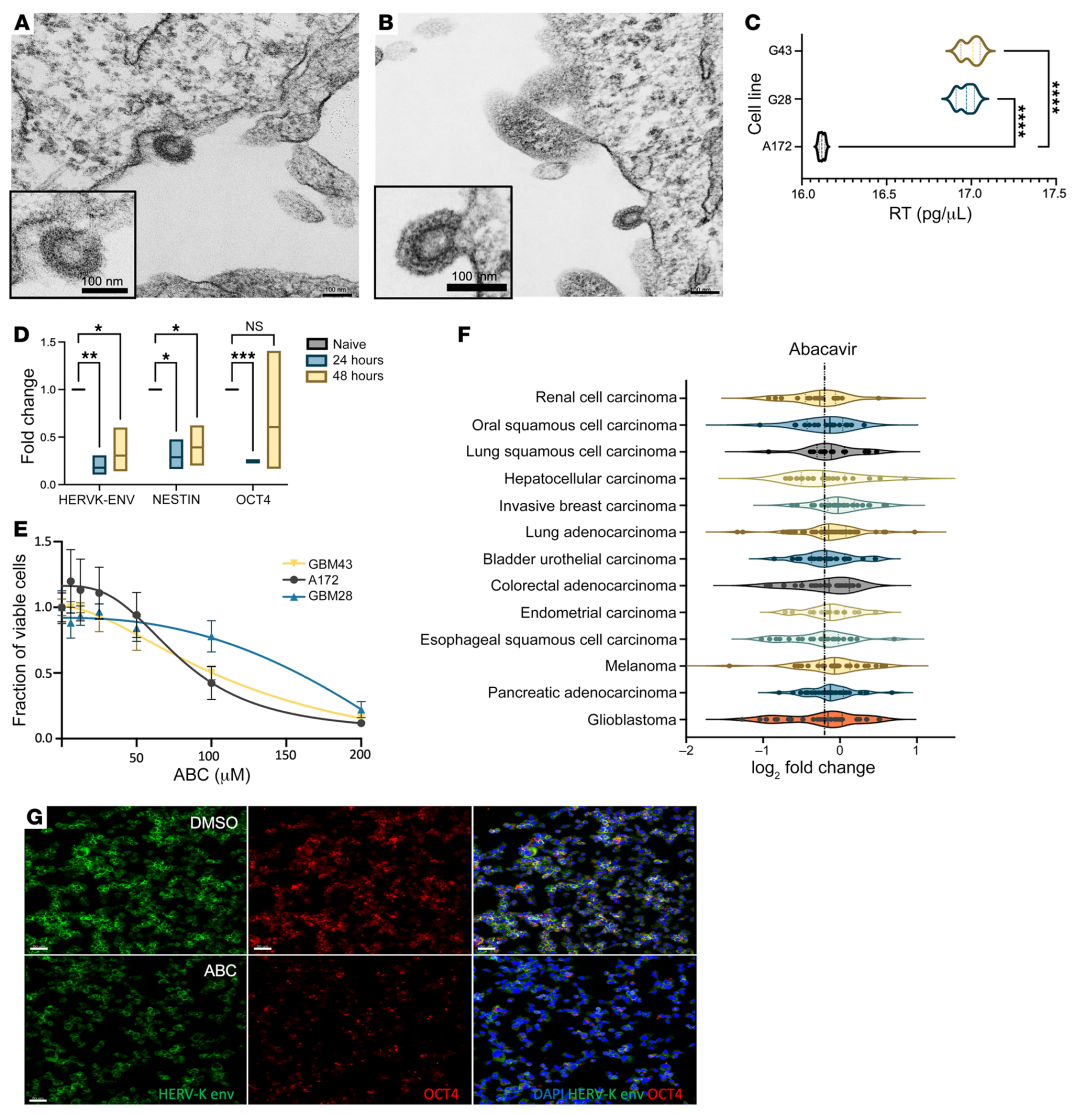
HERV-K viral particles are produced in glioblastoma. (**A**) Transmission electron microscopy demonstrating HML-2 virions budding from HK-transfected 293T cells. Scale bar: 100 nm. Immature virions demonstrate double-membrane morphology with envelope spikes and core capsid proteins (original magnification, ×10,000; ×20,000 [insets]). (**B**) Immature retroviral virions can be seen in naive glioblastoma neurospheres that share similar morphology as positive control-transfected 293T cells. Scale bar: 100 nm. (**C**) Using a PERT assay, reverse transcriptase levels from extracellular vesicles were significantly elevated in HML-2^+^ glioma neurospheres compared with HML-2–deficient glioma cell line A172 (1-way ANOVA, ****P < 0.001). (**D**) Sublethal dosage of abacavir (20 μM) reduced expression of pluripotency markers OCT4 and Nestin at 24 and 48 hours after exposure with qPCR (1-way ANOVA, *P < 0.05, **P < 0.01, ***P < 0.001, ****P < 0.0001). (**E**) Abacavir reduced cell viability of glioma cell lines in a dose-dependent manner (IC_50_ = 75.8–123.1 μM) using an XTT cell viability assay. (**F**) Abacavir (20 μM) reduced HERV-K ENV protein expression and OCT4 at 72 hours in patient-derived glioma neurospheres by immunofluorescence. (**G**) Abacavir inhibited tumor cell proliferation in a variety of cancer cell lines, with notable effect in GBM (median = –0.1550, interquartile range = –0.6601 to 0.02814). Data obtained from the DepMap. Scale bars: 50 μm.

Because near-full-length HERV-K loci were identified by scRNA-Seq at Chr7p22, we sought to determine if our GBM neurospheres derived from patients with GBM were producing HERV-K viral-like particles using transmission electron microscopy. Electron microscopy of 293T cells transfected with the HML-2 consensus sequence demonstrated virions (~100 nm) budding from the plasma membrane (Figure 10A). We discovered active budding of similar retroviral virions in GBM that retained the structural morphology of HML-2. The viral particles possessed prominent envelope spikes with a semihollow capsid core suggestive of an immature virion (Figure 10B). Transmission electron microscopy with immunogold labeling confirmed nuclear sparing and cytoplasmic localization of HERV-K envelope protein within the Golgi vesicles and plasma membrane; furthermore, HML-2 env protein was also detected on plasma membrane microvillous extensions in several cells, suggesting active extracellular export (Supplemental Figure 7). We next isolated extracellular vesicles from patient-derived GBM neurospheres and observed expression of isolated retroviral proteins HML-2 env and reverse transcriptase (RT) (Supplemental Figure 8). Additionally, we observed higher expression of RT in patient-derived GBM neurospheres compared with that in the HML-2–deficient non-stem-like GBM cell line A172 (Figure 10C). Taken together, these findings support the presence of HML-2 retroviral proteins within extracellular vesicles in GBM.

### HML-2 can be targeted with the antiretroviral nucleoside RT inhibitor.

HML-2 can be targeted by antiretroviral drug therapies such as nucleoside RT inhibitors (NRTIs) (38). We evaluated the ability of the commercially available NRTI, abacavir, to abrogate HML-2 proviral expression and cell viability in patient-derived GBM neurospheres. This response was recapitulated in a variety of established cancer cell lines using the Cancer Dependency Map (DepMap) (Figure 10, F and G). We observed decreased transcription and protein expression of HML-2 env and the stem cell marker OCT4 following 72 hours of treatment with abacavir. The Chr7p22 locus we previously identified in our patient-derived cell lines includes an intact pol gene and can produce a functioning RT. Using the CRISPRi HML-2 construct or abacavir treatment to target HML-2 expression, we observed a decrease in the activity of HML-2 RT using a product-enhanced RT (PERT) assay on the media from our transfected cells. The PERT assay was developed to detect retroviral RT activity utilizing native RT enzymes from cellular supernatant (39). Using an RNA template from an MS2 bacteriophage plasmid and a standardized curve for RT activity generated from HIV-1 RT, we identified increased endogenous RT expression in our patient-derived GBM cells as compared with the A172 glioma cell line (39) (Figure 10, C–E). The PERT assay demonstrated a marked decrease in HML-2 RT activity after 48-hour exposure to abacavir and after transfection with the CRISPRi-HML-2 construct (Supplemental Figure 8E).

## Discussion

GBM is the most aggressive primary brain tumor; its median survival is 14 months despite conventional surgical resection and adjuvant chemoradiation (40). Molecular heterogeneity drives treatment resistance and recurrence of high-grade gliomas and is largely dependent on transcriptional subtypes and distinct single-cell programs. Within traditional transcriptomic subtypes of GBM (classical, proneural, and mesenchymal), significant cellular heterogeneity exists that longitudinally evolves with tumor treatment (23, 41–43). These unique cellular drivers determine the transcriptomic fate of these tumors and are enriched with undifferentiated GBM stem cells (25). GBM stem cell states are considered responsible for GBM heterogeneity, tumor propagation, treatment resistance, and parenchymal invasion. Therefore, targeting the GBM stem cell niche is an attractive option to eliminate cellular transitional states and reduce tumor recurrence and treatment resistance.

Here, we have demonstrated for the first time to our knowledge that HML-2 plays a role in defining the stem cell state of high-grade gliomas. Our transcriptomic analysis identified several single-cell states, mainly neural progenitor cells that are enriched with HML-2 viral transcripts. Among the differentially expressed HML-2 loci, 5 loci (3q21.2, 7p22.1, 11q22.1, 8p23.1, 19q11) contained a near-full-length retroviral sequence (gag, pol, env) that can form viral-like particles, as we discovered. Previously, the NPC-like cellular lineage in GBM samples was suggested to play a role in gliomagenesis (26, 27, 44). Several groups have demonstrated that oncogenic transformation and dedifferentiation of glioma precursor cells is reliant on activation of mTOR (45–48). Similarly, our group established that the HML-2 envelope protein interacts with mTOR and CD98HC to maintain pluripotency in neural progenitor cells and regulate neuronal differentiation. Accordingly, epigenetic silencing or HML-2 downregulation reverses stemness and promotes cellular differentiation, suggesting a potential role of HML-2 in gliomagenesis (32).

In gliomas, a bidirectional relationship between HERV-K HML-2 LTR and OCT4 is predicated on the HML-2 LTR OCT4 binding motif. Allosteric inhibition of the HML-2 LTR prevents retroviral transcription but also reduces pluripotency transcripts, while exogenous reintroduction of HERV-K conversely increases OCT4 levels, upregulates stem cell transcriptional pathways, and promotes cellular dedifferentiation and migration. Gene network analysis shows that certain overexpressed genes in GBM tissue relative to normal cortical tissue are associated with maintaining pluripotency of stem cells (Supplemental Figure 9). In astrocyte precursor cells, HML-2 induced significant morphological changes in 3D culture and promoted distant cellular migration, a finding that is a hallmark of glioma invasion in the brain. Previously, Grow et al. uncovered an active binding motif for OCT4 (POU5F1) within the *LTR5Hs* that promoted HERV-K transcription during embryogenesis (30). Additionally, DNA hypomethylation of HERV-K LTRs and OCT4 binding facilitated transcription and production of HERV-K viral-like particles in pluripotent primordial germ cells. Previously, several studies have demonstrated that HERV-K transcription is directly dependent on chromatin remodeling (SMARCB1), histone modification (SETDB1), and DNA methylation (8, 13, 49–51). Our results validate these original findings, ultimately demonstrating that HML-2 drives the glioma-stem cell phenotype through a complex interaction with OCT4.

HML-2 has previously been implicated as a driver of oncogenesis in several cancers. Elevated levels of HML-2 retroviral proteins (GAG and ENV) have been demonstrated in the sera and tumor tissues from patients with breast cancer, prostate cancer, and melanoma (52–55). Our results similarly demonstrate that HERV-K *ENV* RNA and envelope protein are differentially expressed in gliomas compared with control brain tissue from patients with epilepsy. Using RNA-Seq, we similarly demonstrated markedly higher levels of HML-2 coding transcripts compared with controls. CSF also demonstrated a similar trend, suggesting that HERV-K *ENV* RNA could serve as a potential biomarker of disease for gliomas. We also demonstrated that high expression of HML-2 and its protein coding loci, including GAG, POL and ENV, were negative prognostic factors in GBM. Additionally, HERV-K has been detected in the genome of over 90% of samples from Chinese Glioma Genome Atlas and HERV LTRs were differentially upregulated in glioma tissue compared with normal brain samples (56). Other groups have also demonstrated variable expression of full-length envelope HERV-K RNA transcripts in gliomas (21). In this study, we primarily focused on the HML-2 envelope protein because it has been suggested as an oncogenic driver in other cancers. Previously, HERV-K ENV has been associated with increased cellular migration, proliferation, and invasion through the Ras/ERK pathway (16, 57). Aside from the envelope protein itself, HERV-K envelope splice products (np9 and rec) also have been implicated in oncogenesis and have been suggested to mediate cellular invasion and premalignant transformations. Although previous studies have suggested that HML-2 splice products are not actively expressed in human gliomas, our transcriptomic analysis demonstrated some loci capable of forming splice products, rec and np9 (Supplemental Table 5) (21).

A viral etiology for GBM has been proposed by several studies; yet no causative agent has been established (58, 59). Nevertheless, HERV-K reactivation may be a secondary phenomenon in GBM pathogenesis that is precipitated by a primary viral infection such as CMV or EBV. For example, Assinger et al. demonstrated that CMV has been shown to activate HERV-K transcription by dysregulating DNMT1 and -3 (60). Additionally, tumor-related viruses (HTLV-1, HBV, KSHV, and EBV) may also upregulate HERV-K through transactivation of HERV-K LTRs (14). Therefore, this aberrant HERV-K expression may be a viral restriction defense program acquired during human evolution that becomes markedly dysregulated in cancers such as GBM. Hence, it is possible that prior viral insults reactivate HERV-K (HML-2) in glial precursor cells, promoting dedifferentiation and gliomagenesis.

Previously, we have demonstrated that antiretroviral drugs, specifically NRTIs and integrase inhibitors, reduce expression of HML-2 transcripts (38, 61). In cells transfected with pc-HK, NRTIs reduced both RT levels and HML-2 transcripts, respectively (38). Similarly, our data further validate that NRTIs such as abacavir can curtail endogenous HERV-K polymerase and envelope expression, suggesting that these glioma cells demonstrate basal expression of RT. Additionally, abacavir substantially reduced GBM cell viability and improved survival in both GBM and medulloblastoma mouse models (Figure 10G and Supplemental Figure 8H) (62). In the clinical setting, NRTIs have also been shown to improve outcomes in patients with HIV and GBM, compared with those patients not on antiretroviral therapy (63, 64). Notably, we found evidence of immature retroviral virions in our electron microscopy samples and evidence of active subcellular export of HERV-K envelope proteins. It is important to note that the retroviral virion formation in gliomas is likely a rare phenomenon that is likely a hallmark of the cancer stem cell phenotype. Although we did not examine for viral transmission of HERV-K virions in gliomas, HERV-K–mediated intercellular transfer could be a possibility in certain glioma stem-like cells.

Because certain glioma subpopulations express full-length HERV-K transcripts, utilizing antiretroviral therapeutics may be useful in targeting the stem cell niche in GBM. Here, we demonstrate that the brain-penetrant NRTI, abacavir, reduced HERV-K retroviral protein expression at sublethal dosing while concurrently reducing OCT4 expression. Abacavir has also been suggested to reduce proliferation and induce differentiation in malignant medulloblastoma (65, 66). Similarly, Maze et al. demonstrated that HIV protease inhibitors reduced proliferation of HERV-K^+^ schwannomas, and meningiomas by eliminating ERK1/2 tumorigenic pathways (67). Given their favorable pharmacokinetic profile and their specificity for HERV-K, antiretroviral therapeutics may be ideal candidates for cancer drug repositioning.

HERV-K transcriptional activation remains a critical component of the glioma stem cell state. Although normally epigenetically suppressed, HERV-K reactivation occurs in malignant gliomas and is mediated by the pluripotency factor OCT4. Disabling HERV-K transcription impairs glioma tumorigenesis and pluripotency, suggesting that HERV-K may be a viable target for certain subsets of patients with GBM. Future studies assessing the expression of HERV-K within large-scale bioinformatic databases will improve our understanding of the glioma retrotranscriptome and its clinical significance.

## Methods

### Patient samples.

Deidentified patient glioma samples and perilesional CSF were obtained from the NIH Surgical Neurology Branch. Briefly, samples were resected and frozen-embedded in OCT media to preserve RNA and protein quality. During resection, perilesional CSF was also harvested from the resection cavity or adjacent cisterns/sulci. CSF samples were briefly centrifuged to remove any debris prior to storage. Patient demographics and relevant histopathology can be found in Supplemental Table 1.

### Plasmid/DNA constructs.

CRISPR/dCas9-HERV-K plasmid was constructed using a Sin3-repressive interacting domains construct with 4 guide RNAs targeting the HERV-K *LTR5Hs* (Supplemental Table 9). This dead Cas9 construct allosterically inhibits transcriptional activation through chromatin remodeling. Control plasmids contained CRISPR plasmids with off-target RNA sequences (13). The pc-HK plasmid was constructed using a pcDNA3.1 vector (Invitrogen) with an inserted HERV-K consensus sequence as previously described (38).

### Cell culture.

Patient-derived GBM neurospheres (GBM28, IDH WT, 68-year-old male, and GBM43, IDH WT, 69-year-old male) were obtained from the Mayo Clinic Brain Tumor Patient-derived Xenograft National Resource (33) and maintained in serum-free media and DMEM/F12 without phenol red (Invitrogen), supplemented with 20 ng/mL epidermal growth factor and fibroblast growth factor, 2% B27 (Invitrogen), 1% penicillin-streptomycin, and 1% sodium pyruvate (Fisher Scientific). Established cell lines (A172 and normal human astrocytes) were obtained from ATCC and maintained according to manufacturer’s guidelines in their respective media: A172 (DMEM with 5% FBS supplemented with penicillin/streptomycin) and normal human astrocytes (Astrocyte Growth Medium Bullet Kit, Lonza). TrypLE Express (Invitrogen) and Trypsin/EDTA (Sigma-Aldrich) were used to dissociate neurospheres and established cell lines, respectively. For HML-2 transfection assays, CD34^+^ neural stem cells were cultured in astroglial differentiation media with 10% FBS (more than 10 passages). Derived astrocyte precursor cells were transfected with the HML-2 consensus plasmid or a control plasmid using Lipofectamine 3000 (Thermo Fisher Scientific) for 48 hours in low-adhesive vessels. For viability assays, cells were seeded in a 96-well plate at a concentration of 100 cells/μL and treated with abacavir (ARP-4680, NIH HIV Reagent Program) at increasing concentrations (0–200 μM). Cells were incubated for 4 days in 37°C, and at 48 hours they were redosed with abacavir. Cell viability was measured using the quantitative colorimetric tetrazolium dye (XTT) assay kit according to the manufacturer’s instructions (ATCC). Absorbance was measured using a Biotek ELx800 microplate reader at 450 nm. For neurosphere formation assay, neurospheres were transfected with either ngCRISPR/dCas9 or CRISPR/dCas9-HK. 1,000, 500, 250, 125, 60, 30, and 15 cells were seeded in 96-well plates in triplicates for each cell number and the treatment condition. Seven days after transfection, the number of neurospheres formed in each well was plotted against the number of cells seeded.

### RNA-Seq.

Fresh frozen samples from 71 glioma samples were processed for bulk RNA-Seq at the New York Genome Center. Sample RNA from tumor samples and CD34^+^ NSC-derived astroglial cells was extracted using Trizol (Thermo Fischer Scientific), and only samples with high-quality RNA were utilized for analysis (RIN > 7). Samples were prepared for RNA-Seq using the following parameters: stranded, paired end, >100 base pairs, with greater than 50 million reads. Resultant fastq files were aligned to the human genome (hg38) using STAR aligner and profiled with a custom ensemble workflow using TE-Transcripts (from the M. Hammell Laboratory, Cold Spring Harbor Laboratory, Cold Spring Harbor, New York, USA) to produce count matrices of over 50,000 individual HERV loci. A similar workflow was utilized for the raw scRNA-Seq fastq files for over 66,000 cells from the European Genome-Phenome Archive (EGAS00001005434) (24). Because not all HERV-K loci can produce functional proteins, we subsequently filtered our data for 72 HML-2 loci that contained full ORFs for downstream analyses. Differential expression for CD34^+^ NSC-derived astroglial cells was performed using DESeq2 with multiple-testing correction for both 3D and adherent cultures. Functional pathway and gene ontology analysis were visualized using the R packages pheatmap and ggplot. Relevant bulk RNASeq data from our patient cohort is available in a certified collection at Synapse (see *Data availability*).

### Transcriptional state assignment.

For the single-cell data and bulk RNA-Seq data, we utilized a custom script to rank and assign transcriptional states based on their requisite genes derived from an integrative model on cellular states in GBM. For scRNA-Seq, cells were filtered based on percentage of mitochondrial transcript expression (>5% mitochondrial transcripts) as well as the number of detected features (<200 or >2,500 features) to remove low-quality single-cell transcriptomes from our analysis. In addition, doublet cells were removed using the R package DoubletFinder. Filtered scRNA-Seq data were normalized and scaled using the R package Seurat. Scaled normalized counts were then converted to a ranked list for input for gene set enrichment analysis. Ranked lists were then used as input to score individual cells for their expression of transcriptional state signatures using the R package singscore. The transcriptional state identity of each cell was then assigned based on the most highly ranked expression program representing the predominate transcriptional state.

### Analysis of HML-2 expression in transcriptional states.

Output of the transcriptional state assignment script was analyzed in Seurat. Data were normalized and principal component analysis was run on all included loci and genes. Using the Seurat function FindClusters, cells were clustered according to their predominant transcriptional states. The Seurat function FindMarkers was utilized to calculate log_2_ fold change in expression of unique markers of individual clusters. Outputs were produced with adjusted *P* values. ANOVA of overall HML-2 expression across transcriptional states was performed using the Seurat function VlnPlot with multiple comparisons correction. Ridgeplots, dotplots, and feature maps were produced using the Seurat functions RidgePlot, DotPlot, and FeatureMap, respectively. UMAP plots were generated using the Seurat functions RunUMAP and DimPlot. Heatmaps were generated using the Seurat function DimHeatmap and the R packages ggplot and heatmaply.

### RNA in situ hybridization.

Multiplex RNA in situ hybridization was performed using the RNA-scope Multiplex Assay v2 (Advanced Cell Diagnostics) on fixed cells and paraffin-embedded tissue. Briefly, patient-derived glioma neurospheres were plated into chambered slides containing Geltrex and fixed with 4% paraformaldehyde and serially dehydrated/rehydrated prior to probe hybridization. Probes specific for the HML-2 envelope transcripts, *OCT4*, and *Nestin* were established per manufacturer’s recommendation. The HML-2 probe (C1), *OCT4* (C2), and *Nestin* (C3) were serially hybridized in the HybEZ Oven at 40°C using Opal 520, 570, 620 fluorophores. Slides were costained with DAPI mounting media. Bright-field images were acquired using a Leica SP8 confocal microscope (×40 magnification; amber, 670 nm; red, 580 nm; green, 488 nm). Postprocessing for all images was performed using Imaris (Bitplane version 9.3). Negative controls were included per manufacturer protocol (ACD Bio).

### Western blot analysis.

Cells were either treated with abacavir for 48 hours or nucleofected and cultured 72 hours prior to collection. Cells were collected and protein extracted using RIPA buffer (Sigma-Aldrich) with protease/phosphatase inhibitor (Cell Signaling). Protein concentration was measured using Bio-Rad Protein Reagents according to manufacturer’s instructions. Protein samples were then denatured in NuPAGE Reducing Agent (×10) and LDS sample buffer (×4) and then separated in a NuPAGE 4%–12% Bis-Tris gel (Invitrogen) and transferred to nitrocellulose membranes using iBlot transfer device (Thermo Fisher Scientific). Membranes were blocked in 5% omniblock and PBS (Gibco) + Tween (Thermo Fisher Scientific) and then blocked with primary antibodies overnight. Membranes were washed with PBS-T, probed with anti-rabbit or anti-mouse secondary antibodies (Kindle Biosciences), and imaged using ECL chemiluminescence (Kindle Biosciences). Specific antibody information is included in Supplemental Table 10.

### Preparation of cDNA from brain tissue and cell lines.

HML-2 RNA was measured in brain tissue and cell lines. We isolated RNA from tumor tissue and cell line samples using Trizol extraction protocol with chloroform. RNA samples were treated with DNA-ase (Thermo Fisher Scientific) to eliminate residual genomic DNA and diluted in 40 μL nuclease-free water. RNA was quantified using NanoDrop 2000 (Thermo Fisher Scientific), adjusted to a final concentration of 50 ng/μL, and reverse transcribed (SuperScript First-Strand Synthesis RT-PCR kit, Thermo Fisher Scientific). qPCR was then used to amplify and detect target transcripts on the cDNAs using 5 μM primers. Based on our previous work, we utilized qPCR primers specific for HML-2 *ENV* (Supplemental Table 11) based on the consensus sequence for HML-2 from dFam (https://dfam.org/home) and the UCSC Genome Browser using Repeat Masker. For stem cell markers, we used *OCT4* and *Nestin* primers that have been previously validated in our lab. All primer sets were validated for target specificity using in silico PCR (UCSC genome browser, hg38). qPCR cycling conditions were as follows using the Fast SybR green master mix: 95°C for 20 seconds; 95°C for 3 seconds and 60°C for 30 seconds repeated for 40 cycles; 95°C for 20 seconds; and 95°C for 1 second and 60°C for 20 seconds for 40 cycles. Normalized CT values were utilized to quantify expression of target transcripts using the ΔΔCT method. All qPCR runs included RT negative controls that did not amplify. All qPCR experiments were repeated with biologic and technical triplicates.

CSF samples were centrifuged at 300*g* for 10 minutes to remove cells and debris. Total nucleic acids were extracted from 400 μL of the supernatants with an EZ1 Advance XL device (Qiagen) and the EZ1 Virus Mini Kit v2.0 (Qiagen), following the manufacturer’s instructions. Extracted nucleic acids were eluted in 60 μL RNAse-free water, and residual magnetic beads were magnetically extracted.

### Analysis of HML-2 levels by ddPCR.

The ddPCR reaction was set in 96-well plates in duplicate in an AutoDG Droplet Digital PCR System (Bio-Rad) with a set of primers and probe (FAM labeled) to detect HERV-K *env* (forward primer, 5′-ATTTGGTGCCAGGAACTGAG-3′; reverse primer, 5′-GCTGTCTCTTCGGAGCTGTT-3′; and probe 5′-6-FAM-AGGAGTTGCTGATGGCCTCG Iowa Black FQ-3′) (Supplemental Table 12). For the analysis of CSF samples, to confirm the extracellular origin of HML-2 DNA in CSF, a premade assay of primers and probes targeting a cellular DNA (RPP30 gene, HEX-tagged) was also included (Bio-Rad, 10031244). For the analysis of HML-2 RNA in brain tissue and cell lines, *HPRT1* was used as a reference gene (HEX-tagged, Bio-Rad premade assay, 10031256). The master mix was composed of 12.5 μL ddPCR Supermix (no dUTP) (Bio-Rad), 1.25 μL of a mix of HERV-K *env* primers (900 nm) and probe (250 nm) (Bio-Rad), 1.25 μL of RPP30 or HPRT1 assay (Bio-Rad), 2.5 μL of cDNA, and 7.5 μL of RNAse-free water. After preparing the droplets, the PCR was performed in a T100 Thermal cycler (Bio-Rad) with the following cycling conditions: 95°C for 10 minutes, 40 cycles of 95°C for 30 seconds and 60°C for 1 minute, and 95°C for 10 minutes. The number of copies was determined in a QX200 Digital PCR reader (Bio-Rad).

### Determination of HML-2 levels.

HML-2 extracellular DNA levels in the perilesional CSF were expressed as a ratio of HML-2 *env* copies to RPP30 copies (68). HML-2 RNA copies in brain tissue were normalized to the copies of *HPRT1* following previous studies (68, 69). (a) Briefly, the number of copies of *HPRT1* per 25 μL reaction was divided by the theoretical input amount (50 ng), to obtain the “copies of reference gene per theoretical ng of input RNA.” (b) The average reference copies per theoretical ng of input RNA was calculated across all samples to obtain the “average reference value.” (c) Copies of reference gene per theoretical ng of input RNA of each sample were divided by the average reference value to obtain “percentage of average.” (d) Theoretical ng of input RNA was multiplied by the percentage of average to obtain “calculated ng of input RNA.” (e) The number of copies of HML-2 were divided by the calculated ng of input RNA to obtain “HML-2 copies per ng of input RNA.” The ratio of HML-2 copies per ng of input directly correlated with the *HML-2/HPRT-1* ratio.

### Multiplex immunofluorescence.

For efficient high-throughput multiplex imaging of tumor tissue, we utilized a previously validated pipeline for immunofluorescence of human gliomas. We generated a cocktail of 11-well characterized primary antibodies in a single-antibody cocktail mixture. All antibodies have been previously validated to stain their target regions with minimal nonspecific binding and tissue cross-reactivity. Supplemental Table 13 shows the primary and secondary antibodies used for our multiplex immunofluorescence. Briefly, paraffin-embedded tissue slides were deparaffinized and permeabilized using serial xylene and ethanol washes. Antigen retrieval was carried out using a 10 mM sodium citrate buffer (pH 6.0) that was heated for 2 minutes in an 800 W microwave (GE model PEM31DFWW) set at 100% power. After antigen unmasking, slides were blocked using Background buster (Innovex Biosciences, NB306) and FcR blocking solution (Innovex Biosciences, NB309). Subsequently, sections were incubated with the 11-plex primary mixture cocktail for 60 minutes at room temperature and washed multiple times (3 times with dH_2_O) followed by secondary antibody incubation (washed 3 times with PBS and 3 times with dH_2_O). Tissue sections were then counterstained with 1 μg/mL DAPI (Thermo Fisher Scientific) for a reference channel for pixel-pixel registration. Slides were the imaged using an Axio Imager.Z2 scanning fluorescence microscope (Carl Zeiss) equipped with a 20×, 0.8 NA Plan-Apochromat (Phase-2) nonimmersion objective (Carl Zeiss) and a 16-bit ORCA-Flash 4.0 sCMOS digital camera (Hamamatsu Photonics). Each labeling antibody was sequentially captured at their distinct wavelength and digitized individually using ZEN2 image software (16-bit). To quantify HERV-K expression, we used an unbiased artificial intelligence algorithm to randomly define multiple ROIs on our whole-slide immunofluorescence slides (Supplemental Figure 10). Using automated ROI fluorescence quantification, we quantified colocalization of HERV-K and associated stem cell markers (OCT4, Nestin, Sox2, Vimentin) in stem cell–rich ROIs. For automated fluorescent image analysis, multiple voxels were randomly generated and distinct colors were isolated from the reference voxel (Supplemental Figure 10). These single-colored images were converted to 8-bit TIFF files and exported for automatic segmentation and analysis. Automatic segmentation of whole-slide multiplex immunofluorescence images was analyzed using Nikon NIS Elements AR software, focusing on staining intensity in the region of interest. Each voxel was smoothened and clean filtered; stains were captured using intensity of between 8 and 705 pixels. Percentage field coverage in each image was normalized by coverage area in each field. Pearson correlations and heatmaps were generated for each representative spectra in each patient slide using R and GraphPad PRISM.

### PERT assay.

The PERT assay was performed utilizing a previously adopted protocol for cellular RT levels. Cell culture supernatant was centrifuged and filtered to remove cellular debris. Once cleared, the supernatant was exposed to 0.25 mM EDTA and 0.25% Triton-X to release endogenous RT from extracellular vesicles. The template for the PERT assay was created using bacteriophage MS2 genomic RNA that was annealed to an MS2 probe. qPCR was then performed using MS-2 forward, probe, and reverse primers using Applied Biosystems Vii 7. RT levels were expressed as pg/mL, utilizing a standard curve generated from HIV-1 RT.

### Electron microscopy.

GBM neurospheres were plated at high density in a 6-well plate (5 × 10^5^ to 1 × 10^6^ cells per well) and fixed with 4% paraformaldehyde with 0.1% glutaraldehyde for immunogold labeling. After fixation, cells were labeled with HERV-K envelope primary antibody (1:250) and immunogold rabbit anti-mouse antibody (1:250). For ultrastructural preservation, cells were fixed with 4% glutaraldehyde in 0.1 M cacodylate buffer. Slides were silver enhanced and prepared for transmission electron microscopy (JEOL 1200 EXII Transmission Electron Microscope).

### Intracranial orthotopic patient-derived xenografts.

Patient-derived GBM neurospheres transfected with either ngCRISPR/dCas9 or CRISPR/dCas9-HERV-K were cultured for 72 hours and harvested for implantation. 2 × 10^5^ cells were resuspended in 2 μL PBS and implanted into the right frontal lobe of nude athymic mice using the following coordinates (AP, 1.5 mm; DV, 3 mm; ML, 2 mm). Mice were obtained from The Jackson Laboratory. For abacavir-treated mice, intracranial ALZET pumps were implanted into the tumor cavity at day 5 filled with either saline or abacavir (12.3 mg/kg/d). Mice were observed over the course of 4 weeks and sacrificed when moribund. Brains were harvested immediately after sacrifice, fixed with 4% paraformaldehyde, and sectioned for histopathology.

### DepMap cell line analysis.

Data were taken from the DepMap PRISM Repurposing Primary Screen to evaluate the effect of abacavir on tumor cell growth (70). Established cell lines from a variety of cancers were used, with analysis being limited to cancers that had 10 or more cell lines treated with Abacavir. Violin plots with median and quartile ranges relative to log_2_ fold change were visualized using GraphPad Prism.

### ChIP.

Naive GBM28 and GBM43 (1.5 × 10^7^ cells) were cross-linked with 1% PFA in serum-free media. To stop the crosslink reaction, glycine was added to a final concentration of 0.125 M. Chromatin was sheared by sonication and immunoprecipitated with 5 μg anti-RNA polymerase II, Mouse Mab IgG, and anti-human OCT4 overnight at 4°C. Subsequently, immunoprecipitated samples were exposed to proteinase K, and DNA was extracted using a phenol-chloroform protocol. Purified DNA was quantified using a spectrophotometer (NanoDrop, Thermo Fisher Scientific) and analyzed as percentage input in a qPCR reaction using primers specific for the HERV-K LTR5s (LTR Transcription Start Site) and a control genomic gene hypoxanthine phosphoribosyltransferase 1 (*HPRT1*). For specific methodology and buffers used, see Supplemental Methods.

### Statistics.

All experiments were conducted with biological replicates or triplicates and confirmed with technical triplicates. Data are shown as mean ± SEM. Full details for statistics are detailed within each subsection and within Supplemental Table 14. Tests used include unpaired 2-tailed *t* test, Mann-Whitney test, 1-way ANOVA, and log-rank (Kaplan-Meier). *P* values of less than 0.05 were considered significant.

### Study approval.

After NIH Institutional Review Board approval (protocol 03N0164), deidentified patient glioma samples and perilesional CSF were obtained from the NIH Surgical Neurology Branch. All animal procedures were approved by the NIH Animal Care and Use Committee (Animal Study ASP 1503).

### Data availability.

Deidentified RNA-Seq data will be made available from the corresponding author upon request and signature of data transfer agreement. 

## Author contributions

AHS and AN were involved in study initiation and design. AHS, SRR, TTDO, VG, CD, TW, LA, YZ, YKBS, SW, DM, MGM, RKS, MHL, KAZ, JS, AGE, JC, DS, JD, WL, KJ, MEI, RJK, MRG, JD, and AN contributed to conducting experiments, data collection, and analysis. AHS prepared the manuscript, which was reviewed and approved by all authors. AN supervised the work in its entirety.

## Supplementary Material

Supplemental data

Supplemental video 1

Supporting data values

## Version 1 07/03/2023

Electronic publication
