# Supplementary material for: Human endogenous retrovirus K contributes to a stem cell niche in glioblastoma
Source: J Clin Invest. 2023 Jul 3;133(13):e167929. doi: 10.1172/JCI167929 (PMC10313366; doi:10.1172/JCI167929)
Supplement: Supplemental data [file jci-133-167929-s215.pdf]

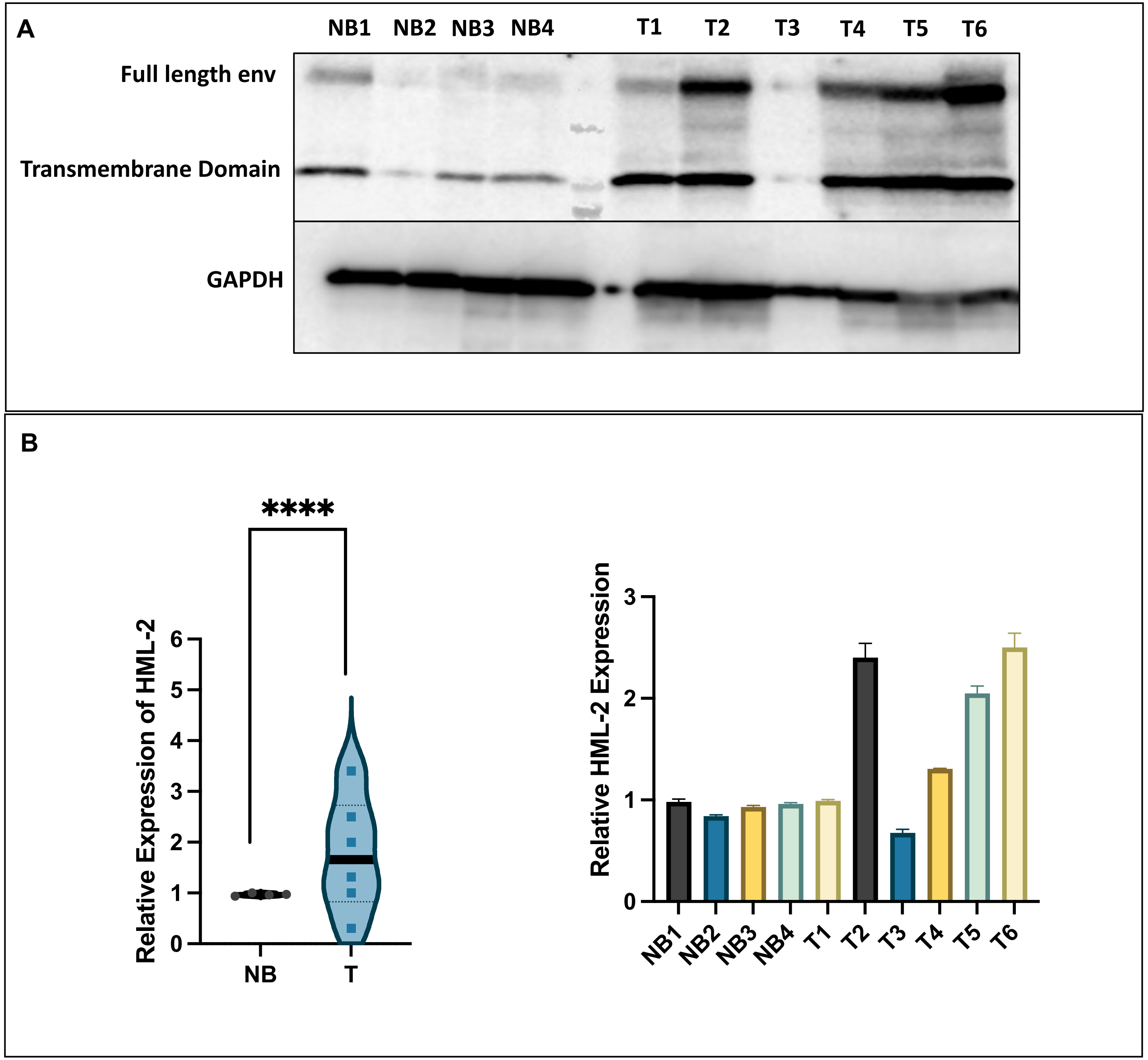
A
NB1 NB2 NB3 NB4 T1 T2 T3 T4 T5 T6
Full length env
Transmembrane Domain
GAPDH
B
****
Relative Expression of HML-2
6
5
4
3
2
1
0
NB
T
Relative HML-2 Expression
3
2
1
0
NB1 NB2 NB3 NB4 T1 T2 T3 T4 T5 T6

Supplemental Figure 1. HML-2 ENV protein is overexpressed in high-grade gliomas. A) Western blot of paired tumor and normal brain tissue shows increased expression of HML-2 ENV protein in high-grade glioma samples. B) Normalized Densitometry demonstrates statistically significant increased expression in glioma patients compared to matched normal brain specimens (p-value<0.0001, two-tailed t-test). Matched normal brain tissue group shows no significant difference in expression (p>0.05). T1=Recurrent GBM, T2=Newly Diagnosed GBM, T3=IDHm recurrent anaplastic astrocytoma, T4=Newly Diagnosed GBM, T5=Newly Diagnosed GBM, T6=Newly Diagnosed GBM.

G

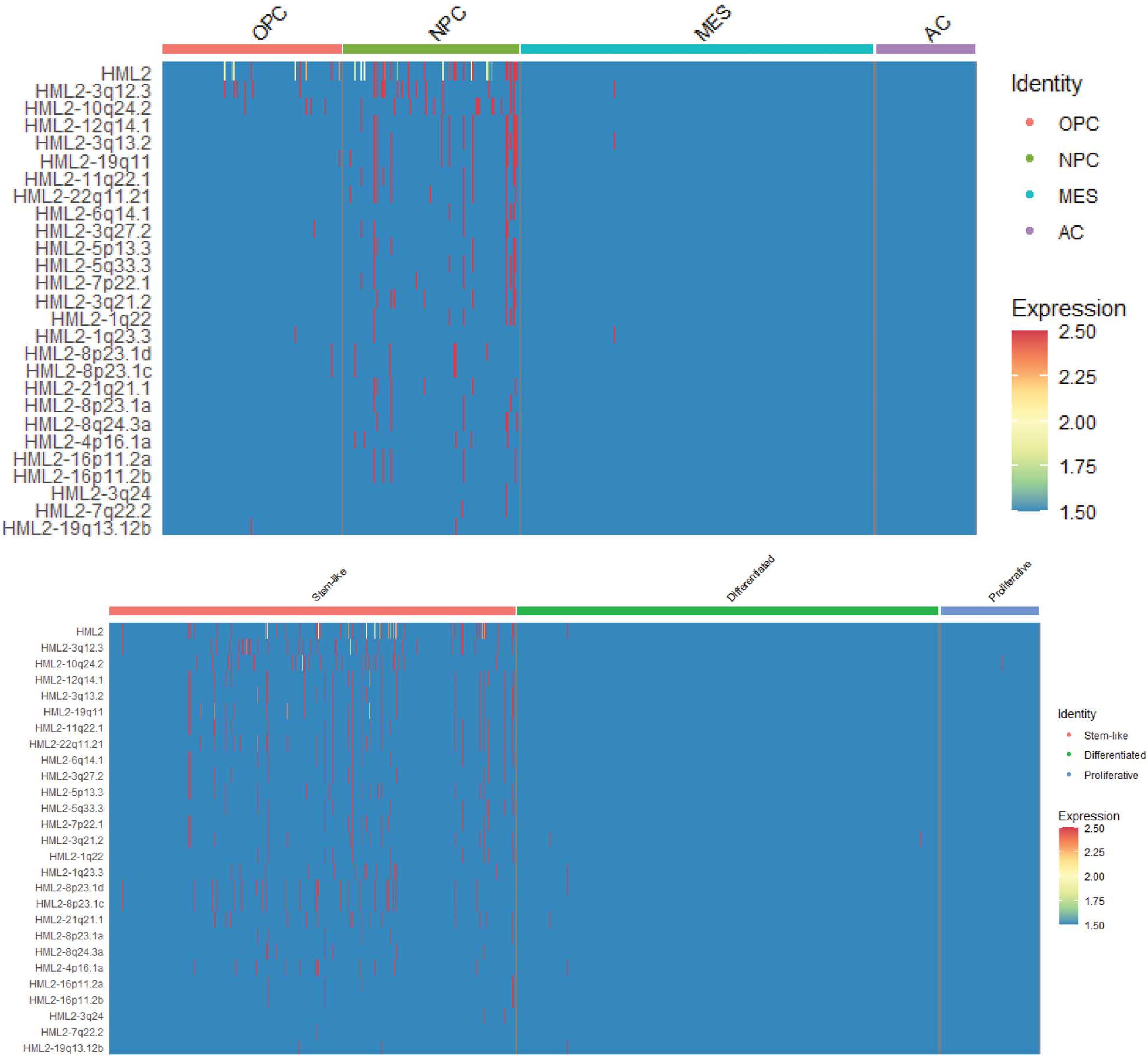
OPC
NPC
MES
AC
HML2
HML2-3q12.3
HML2-10q24.2
HML2-12q14.1
HML2-3q13.2
HML2-19q11
HML2-11q22.1
HML2-22q11.21
HML2-6q14.1
HML2-3q27.2
HML2-5p13.3
HML2-5q33.3
HML2-7p22.1
HML2-3q21.2
HML2-1q22
HML2-1q23.3
HML2-8p23.1d
HML2-8p23.1c
HML2-21q21.1
HML2-8p23.1a
HML2-8q24.3a
HML2-4p16.1a
HML2-16p11.2a
HML2-16p11.2b
HML2-3q24
HML2-7q22.2
HML2-19q13.12b
Identity
OPC
NPC
MES
AC
Expression
2.50
2.25
2.00
1.75
1.50
Stem-like
Differentiated
Proliferative
HML2
HML2-3q12.3
HML2-10q24.2
HML2-12q14.1
HML2-3q13.2
HML2-19q11
HML2-11q22.1
HML2-22q11.21
HML2-6q14.1
HML2-3q27.2
HML2-5p13.3
HML2-5q33.3
HML2-7p22.1
HML2-3q21.2
HML2-1q22
HML2-1q23.3
HML2-8p23.1d
HML2-8p23.1c
HML2-21q21.1
HML2-8p23.1a
HML2-8q24.3a
HML2-4p16.1a
HML2-16p11.2a
HML2-16p11.2b
HML2-3q24
HML2-7q22.2
HML2-19q13.12b
Identity
Stem-like
Differentiated
Proliferative
Expression
2.50
2.25
2.00
1.75
1.50

Supplemental Figure 2. Twenty-six unique HML-2 loci were identified that were uniquely enriched in NPC and stem cell-like populations.

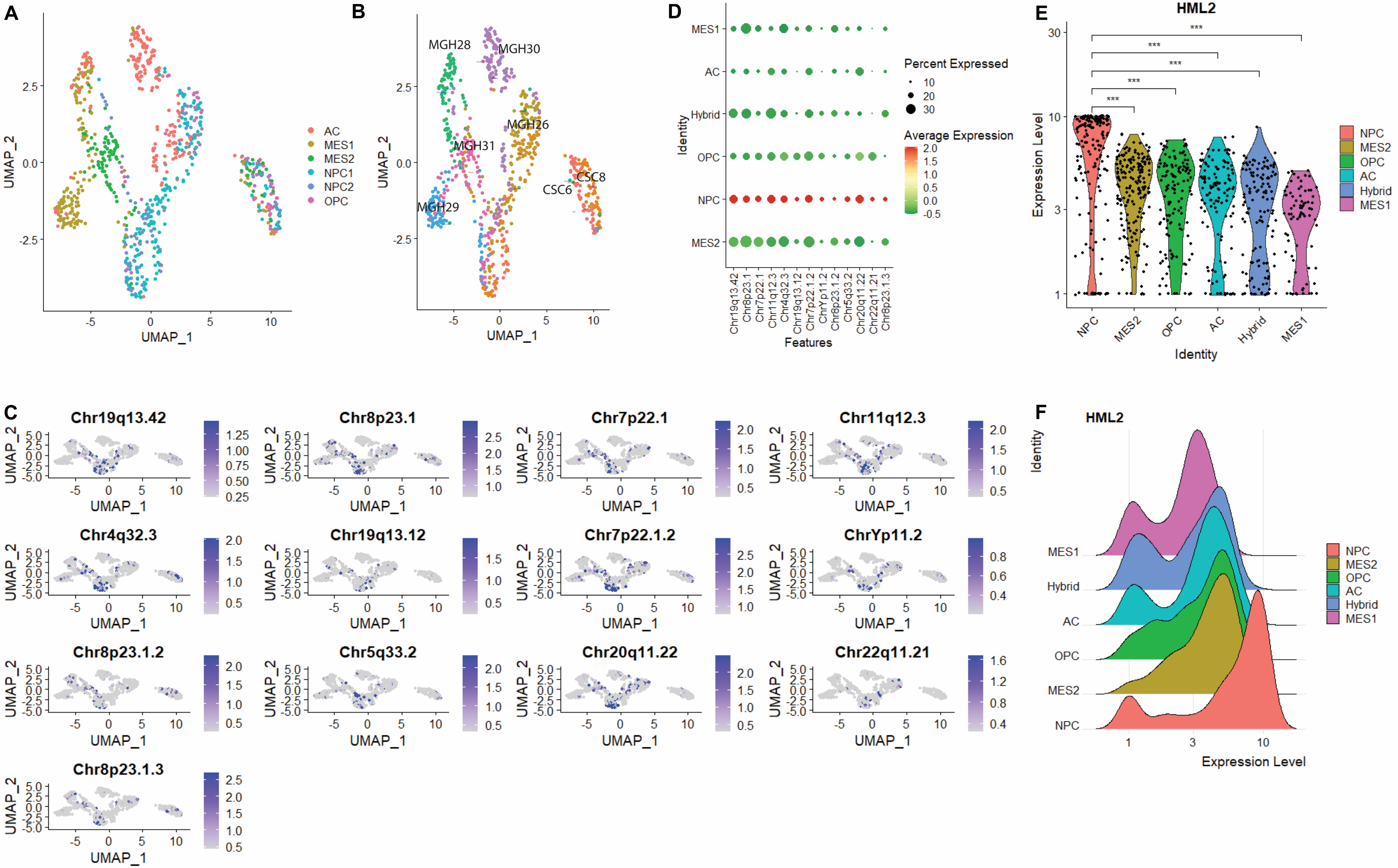

A
B
C
D
E
F
AC
MES1
MES2
NPC1
NPC2
OPC
MGH28
MGH30
MGH26
MGH31
MGH29
CSC6
CSC8
UMAP_1
UMAP_2
Percent Expressed
Average Expression
Identity
Features
HML2
Expression Level
NPC
MES2
OPC
AC
Hybrid
MES1
Chr19q13.42
Chr8p23.1
Chr7p22.1
Chr11q12.3
Chr4q32.3
Chr19q13.12
Chr7p22.1.2
ChrYp11.2
Chr8p23.1.2
Chr5q33.2
Chr20q11.22
Chr22q11.21
Chr8p23.1.3

Supplemental Figure 3. HML-2 is differentially expressed in Neural Precursor-Like Cells. A-B) Unsupervised cluster analysis all HML-2 loci distinguish subpopulations of GBM cellular states, independent of parent tumor origin. C-D) Thirteen HML-2 loci (Chr19q13.42, Chr8p23.1-3, Chr7p22.1-2, Chr11q12.3, Chr4q32.3, Chr19q13.12, ChrYp11.2, Chr5q33.2, Chrq11.22, Chr22q11.21) demonstrated significant enrichment with NPC-like subpopulations, included two loci with tandem repeats at Chr7p22 and Chr8p23. Within our NPC subpopulations, we identified five differentially expressed HERV-K loci with preserved LTR5Hs: *ERV-3697915 (7p22) ERV-2427227(22q11), ERV-610406(11q12.3),ERV_2925064(4q32.3), ERV-3697923(7p22.1).* One loci contains a near-full-length provirus with all coding genes (ERV_3697915,7p22) that map to cell subpopulations in our dataset. E-F) Unsupervised clustering analysis for summary expression of all HML-2 ORF loci distinguishes NPC-like cellular subpopulations independent of tumor origin. (ANOVA, multiple-testing correction, p<0.001).

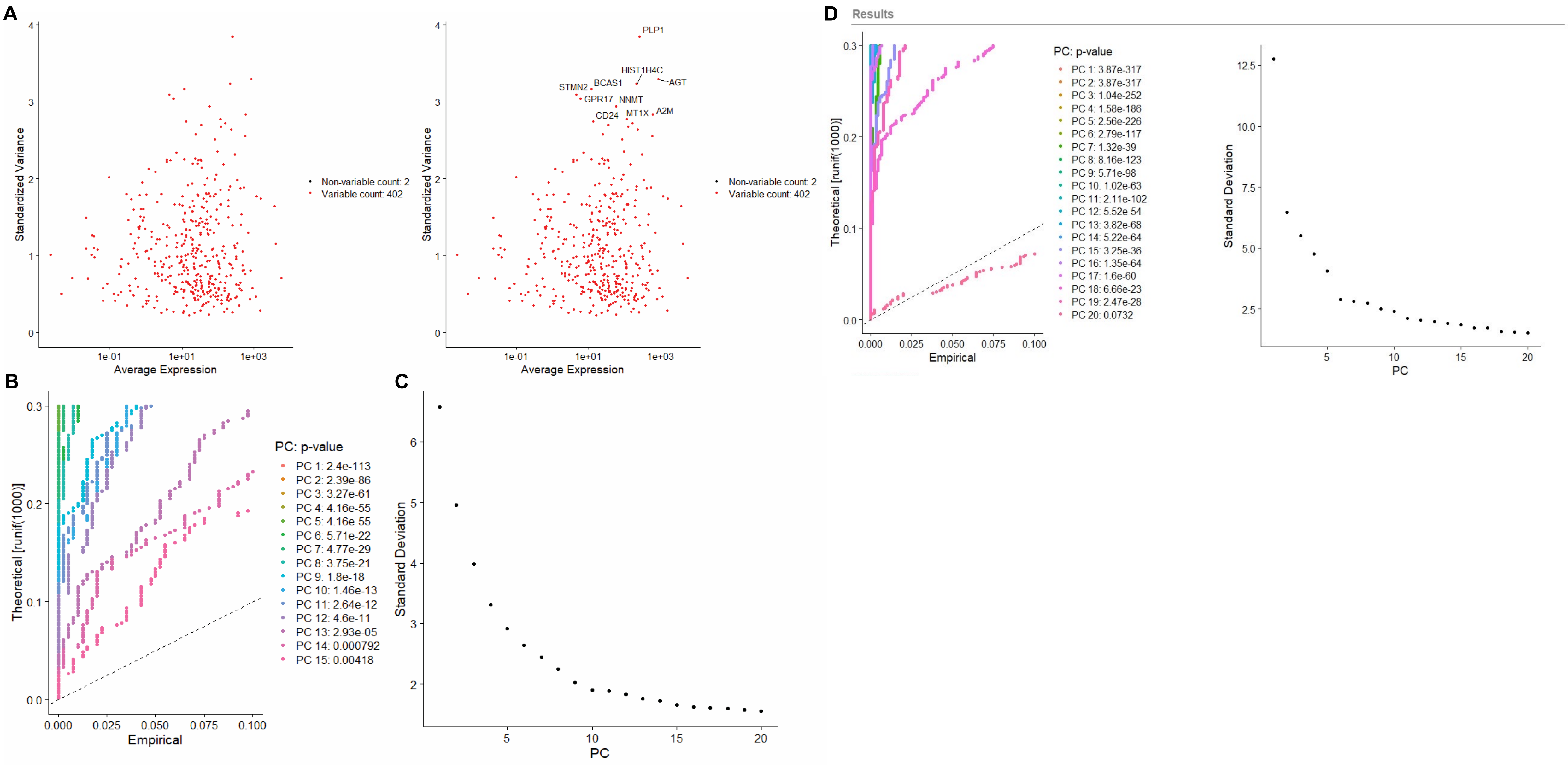
A
Standardized Variance
Average Expression
Non-variable count: 2
Variable count: 402
PLP1
HIST1H4C
AGT
STMN2
BCAS1
GPR17
NNMT
CD24
MT1X
A2M
B
Theoretical [runif(1000)]
Empirical
PC: p-value
PC 1: 2.4e-113
PC 2: 2.39e-86
PC 3: 3.27e-61
PC 4: 4.16e-55
PC 5: 4.16e-55
PC 6: 5.71e-22
PC 7: 4.77e-29
PC 8: 3.75e-21
PC 9: 1.8e-18
PC 10: 1.46e-13
PC 11: 2.64e-12
PC 12: 4.6e-11
PC 13: 2.93e-05
PC 14: 0.000792
PC 15: 0.00418
C
Standard Deviation
PC
D
Results
Theoretical [runif(1000)]
Empirical
PC: p-value
PC 1: 3.87e-317
PC 2: 3.87e-317
PC 3: 1.04e-252
PC 4: 1.58e-186
PC 5: 2.56e-226
PC 6: 2.79e-117
PC 7: 1.32e-39
PC 8: 8.16e-123
PC 9: 5.71e-98
PC 10: 1.02e-63
PC 11: 2.11e-102
PC 12: 5.52e-54
PC 13: 3.82e-68
PC 14: 5.22e-64
PC 15: 3.25e-36
PC 16: 1.35e-64
PC 17: 1.6e-60
PC 18: 6.66e-23
PC 19: 2.47e-28
PC 20: 0.0732
Standard Deviation
PC

Supplemental Figure 4. A) Exploratory analysis of variable genes in our dataset. *PLP1, HIST14C, AGT, BCAS1, STMN2, GPR17, NMNT, CD24, MT1X, A2M*. B) Distribution of p-values for individual principal components relative to uniform distribution (dashed line) C) Percentage of variance explained by individual principal components. The notable inflection point at 10 PC's, suggests that the majority of true signal is explained within the first 10 PC's.

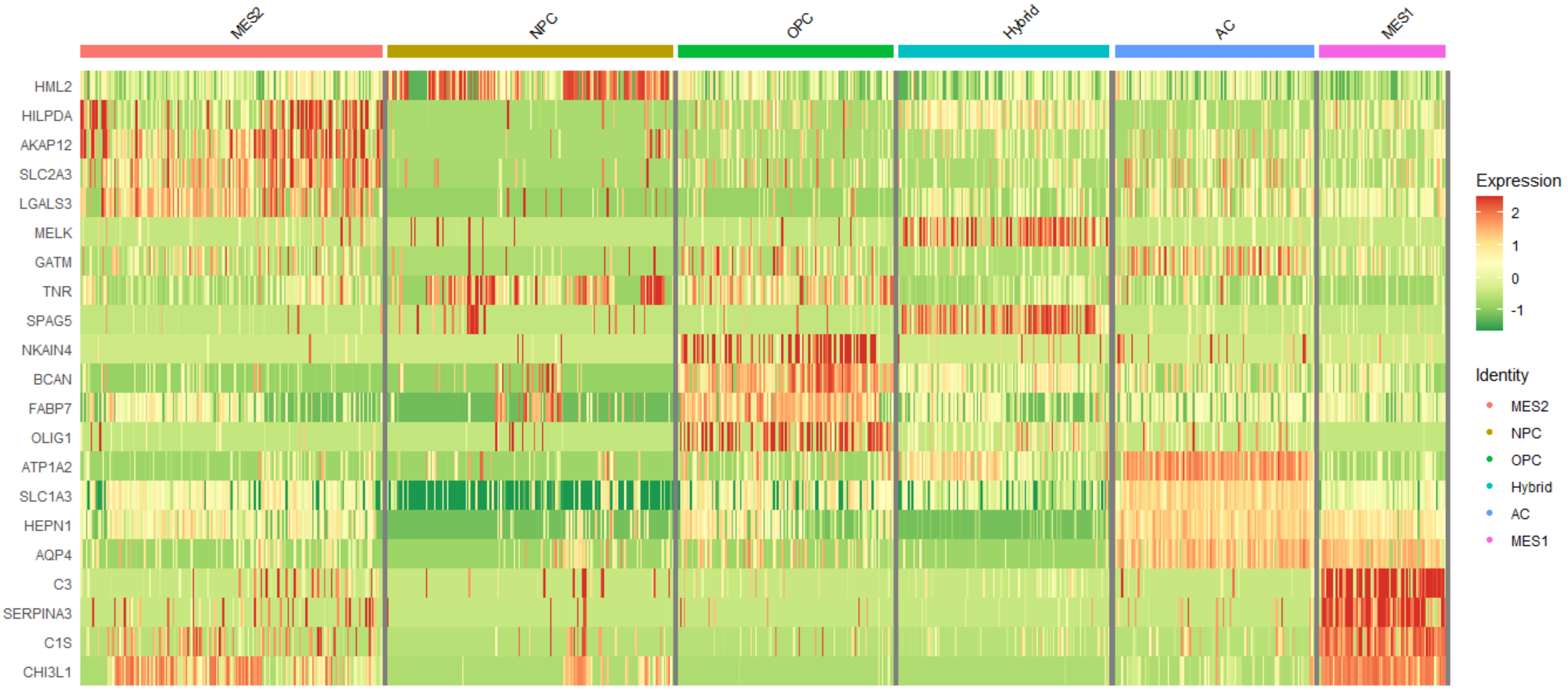

MES2
NPC
OPC
Hybrid
AC
MES1
HML2
HILPDA
AKAP12
SLC2A3
LGALS3
MELK
GATM
TNR
SPAG5
NKAIN4
BCAN
FABP7
OLIG1
ATP1A2
SLC1A3
HEPN1
AQP4
C3
SERPINA3
C1S
CHI3L1
Expression
2
1
0
-1
Identity
MES2
NPC
OPC
Hybrid
AC
MES1

Supplemental Figure 5. Heatmap of differentially expressed genes with respect to cluster identity. Relative expression of HML-2 ORF Loci is highly expressed within NPC-like cells.

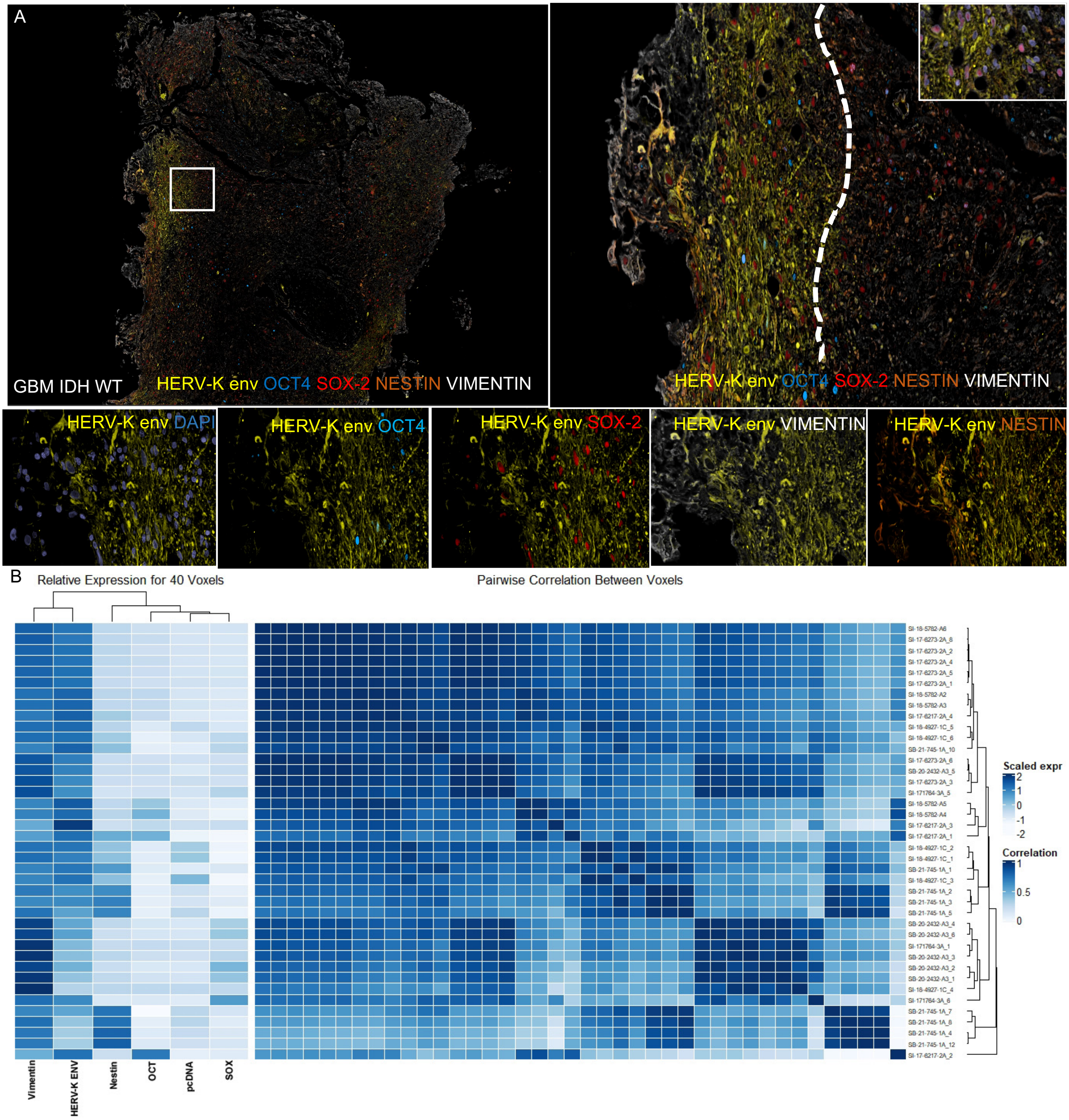
A
GBM IDH WT
HERV-K env OCT4 SOX-2 NESTIN VIMENTIN
HERV-K env OCT4 SOX-2 NESTIN VIMENTIN
HERV-K env DAPI
HERV-K env OCT4
HERV-K env SOX-2
HERV-K env VIMENTIN
HERV-K env NESTIN
B
Relative Expression for 40 Voxels
Pairwise Correlation Between Voxels
Vimentin
HERV-K ENV
Nestin
OCT
pcDNA
SOX
SI-18-5782-A6
SI-17-6273-2A_8
SI-17-6273-2A_2
SI-17-6273-2A_4
SI-17-6273-2A_5
SI-17-6273-2A_1
SI-18-5782-A2
SI-18-5782-A3
SI-17-6217-2A_4
SI-18-4927-1C_5
SI-18-4927-1C_6
SB-21-745-1A_10
SI-17-6273-2A_6
SB-20-2432-A3_5
SI-17-6273-2A_3
SI-171764-3A_5
SI-18-5782-A5
SI-18-5782-A4
SI-17-6217-2A_3
SI-17-6217-2A_1
SI-18-4927-1C_2
SI-18-4927-1C_1
SB-21-745-1A_1
SI-18-4927-1C_3
SB-21-745-1A_2
SB-21-745-1A_3
SB-21-745-1A_5
SB-20-2432-A3_4
SB-20-2432-A3_6
SI-171764-3A_1
SB-20-2432-A3_3
SB-20-2432-A3_2
SB-20-2432-A3_1
SI-18-4927-1C_4
SI-171764-3A_6
SB-21-745-1A_7
SB-21-745-1A_8
SB-21-745-1A_4
SB-21-745-1A_12
SI-17-6217-2A_2
Scaled expr
2
1
0
-1
-2
Correlation
1
0.5
0

Supplemental Figure 6. HML-2 protein expression correlates to expression of stem-cell markers. (A). Using 11-plex whole slide immunofluorescence, glioma stem-cell markers and HERV-K localized to stem-cell rich areas containing abundant OCT4, Sox2, Nestin, and Vimentin expression in IDH wild-type Glioblastoma. (B). Using automated quantification algorithm, cross-sectional expression of HERV-K envelope protein, OCT4, Sox2, Nestin, PCNA, and Vimentin demonstrated significant correlations across randomly generated voxels.

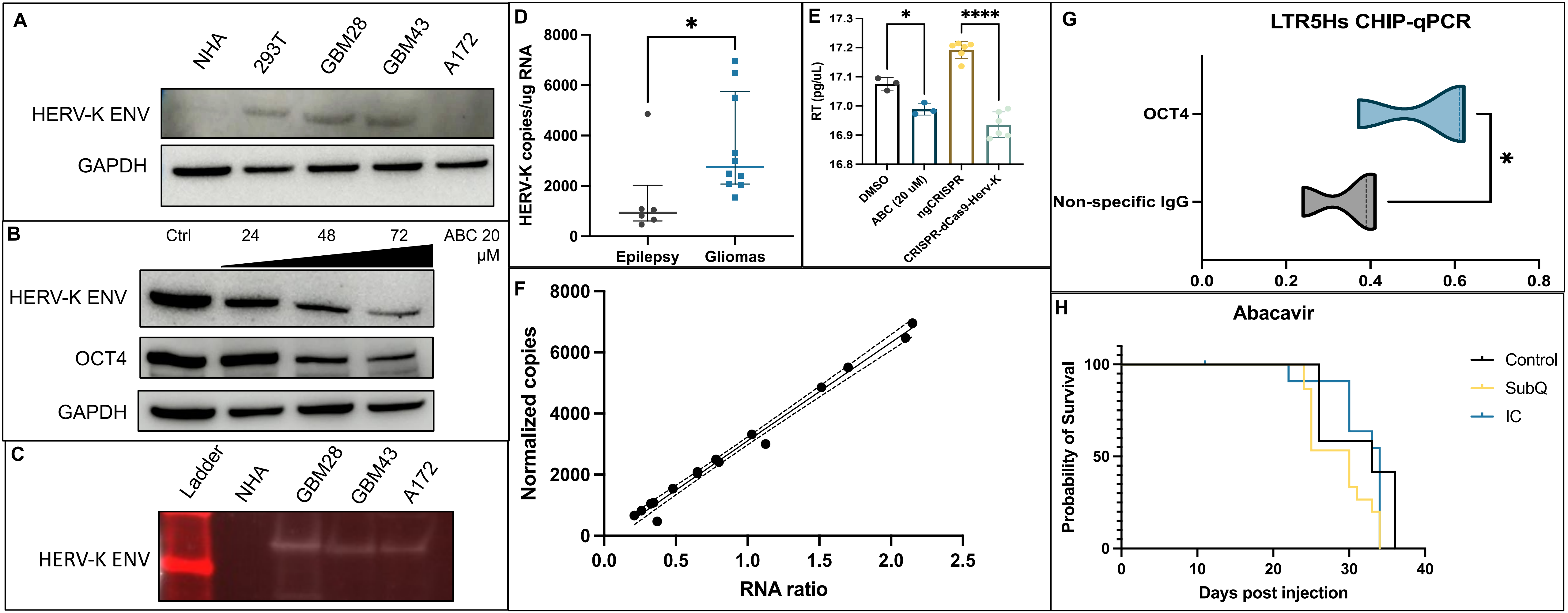

A
NHA
293T
GBM28
GBM43
A172
HERV-K ENV
GAPDH
B
Ctrl
24
48
72
ABC 20 µM
HERV-K ENV
OCT4
GAPDH
C
Ladder
NHA
GBM28
GBM43
A172
HERV-K ENV
D
HERV-K copies/ug RNA
8000
6000
4000
2000
0
*
Epilepsy
Gliomas
E
RT (pg/uL)
17.3
17.2
17.1
17.0
16.9
16.8
*
****
DMSO
ABC (20 uM)
ngCRISPR
CRISPR-dCas9-Herv-K
F
Normalized copies
8000
6000
4000
2000
0
0.0
0.5
1.0
1.5
2.0
2.5
RNA ratio
G
LTR5Hs CHIP-qPCR
OCT4
Non-specific IgG
*
0.0
0.2
0.4
0.6
0.8
H
Abacavir
Probability of Survival
100
50
0
0
10
20
30
40
Days post injection
Control
SubQ
IC

Supplemental Figure 7. A) Western Blot demonstrating that HERV-K ENV is preferentially expressed in Glioblastoma Neurospheres (GBM28 and GBM43) and not in normal human astrocytes (NHA). B)Western Blot demonstrating reduced HERV-K ENV and OCT4 protein expression over 72 hours with exposure to 20 uM of abacavir. C)Extracellular vesicles containing HERV-K ENV can be isolated from the supernatant of Glioblastoma Cells. . D) Normalized copies of HERV-K env RNA in glioma samples is increased compared to control epilepsy samples ($p<0.011$, Mann-Whitney test). E)Treatment with Abacavir (20 uM) and CRISPR-dCas9 reduces extracellular levels of reverse transcriptase compared to DMSO ($p<0.05$, t-test) and ngCRISPR respectively ($p<0.0001$, t-test). PERT was performed as biological triplicate. F) HERV-K RNA ratio directly correlates to normalized copies of HERV-K/ug of RNA. G) CHIP-qPCR validates OCT4 binding to HERV-K LTR5Hs in high HERV-K cell line GBM28 compared to non-specific IgG control. No significant change was noted in the GBM43 cell line with correspondingly less endogenous OCT4 expression. H) Local delivery of abacavir improves survival compared to subcutaneous delivery of abacavir and local saline-treated group in patient-derived murine xenograft model($p <0.03$, Mantel-Cox Test).

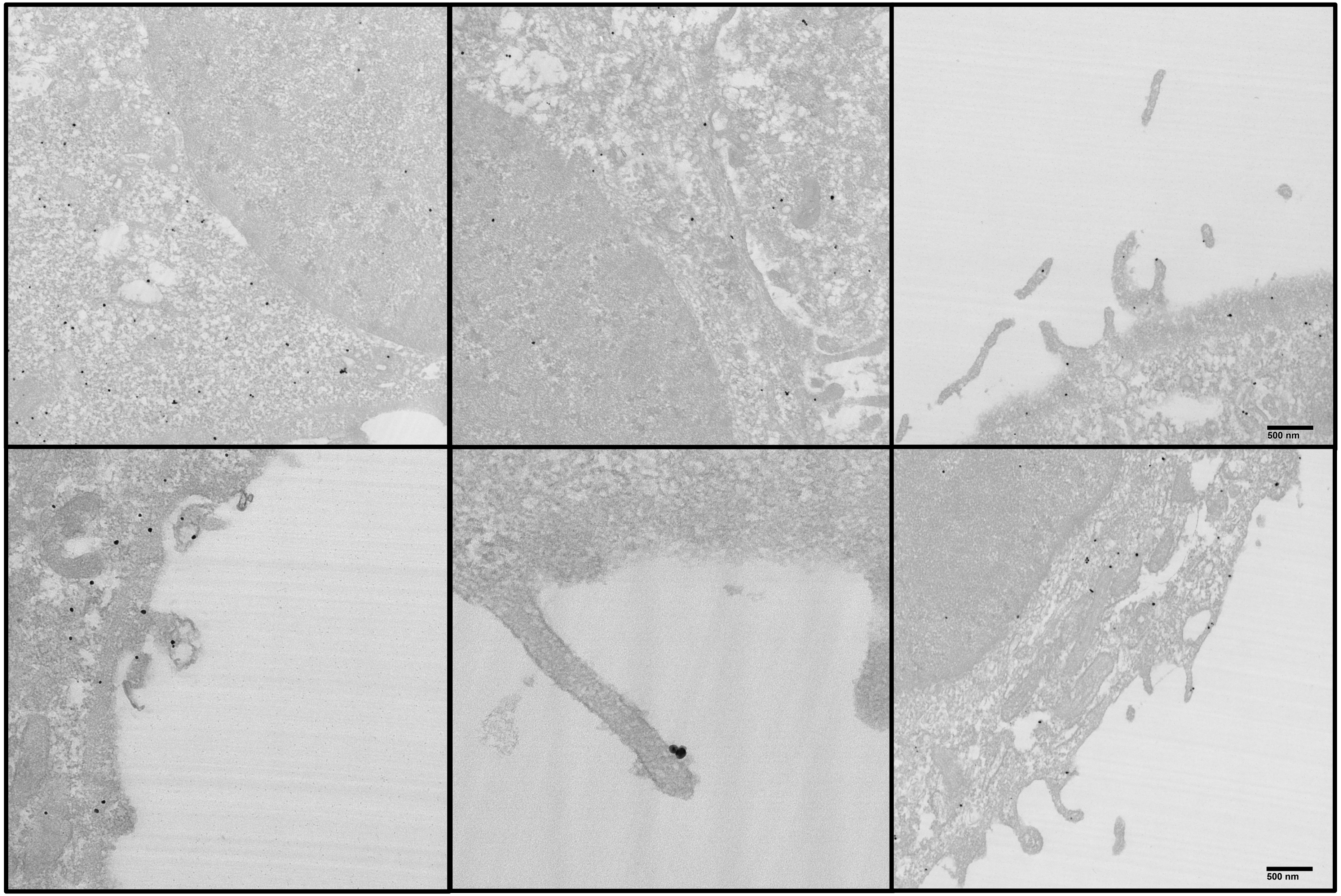

500 nm
500 nm

Supplemental Figure 8. Transmission Electron Microscopy reveals evidence of perinuclear HERV-K envelope expression. Additionally, HERV-K envelope protein can be found in the Golgi vesicles and within microvillous plasma membrane extensions in patient-derived glioma cells. Images taken at 10000X magnification

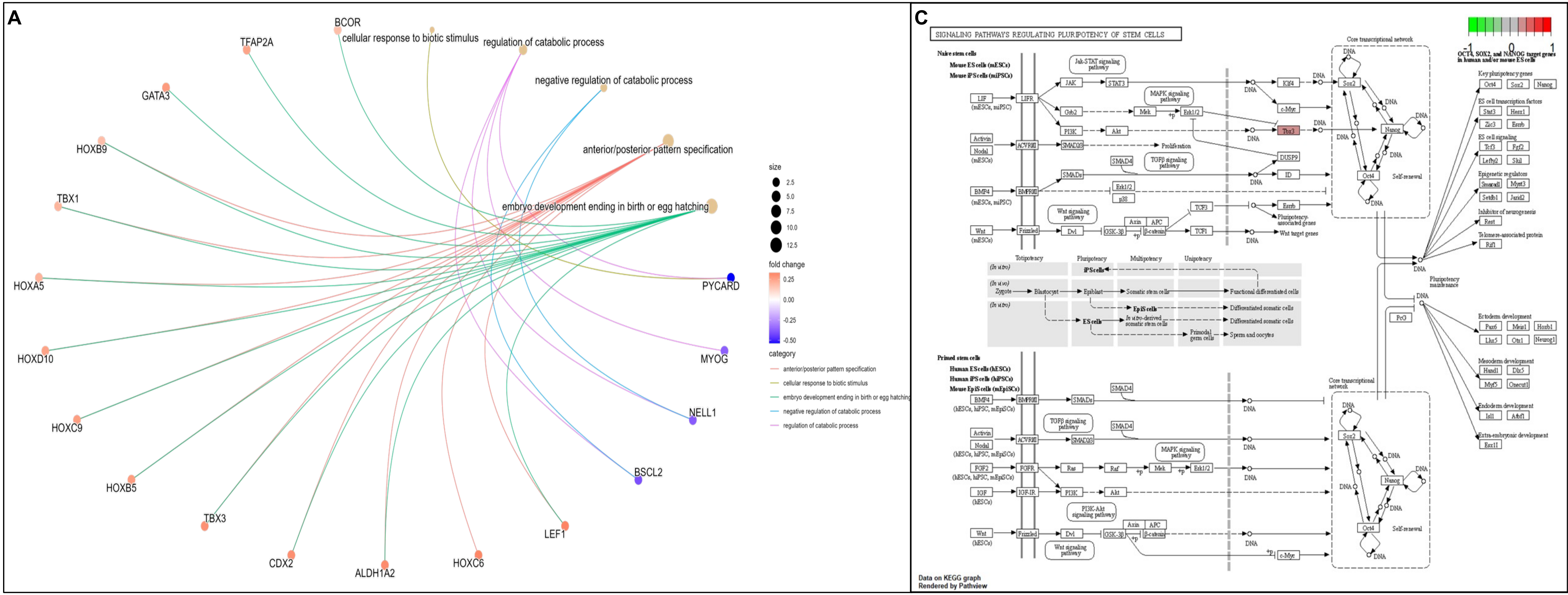
A
BCOR
TFAP2A
GATA3
HOXB9
TBX1
HOXA5
HOXD10
HOXC9
HOXB5
TBX3
CDX2
ALDH1A2
HOXC6
LEF1
BSCL2
NELL1
MYOG
PYCARD
cellular response to biotic stimulus
regulation of catabolic process
negative regulation of catabolic process
anterior/posterior pattern specification
embryo development ending in birth or egg hatching
size
2.5
5.0
7.5
10.0
12.5
fold change
0.25
0.00
-0.25
-0.50
category
anterior/posterior pattern specification
cellular response to biotic stimulus
embryo development ending in birth or egg hatching
negative regulation of catabolic process
regulation of catabolic process
C
SIGNALING PATHWAYS REGULATING PLURIPOTENCY OF STEM CELLS
-1
0
1
OCT4, SOX2, and NANOG target genes in human and/or mouse ES cells
Naive stem cells
Mouse ES cells (mESCs)
Mouse iPS cells (miPSCs)
Primed stem cells
Human ES cells (hESCs)
Human iPS cells (hiPSCs)
Mouse EpiS cells (mEpiSCs)
Core transcriptional network
Self-renewal
Pluripotency maintenance
Key pluripotency genes
ES cell transcription factors
ES cell signaling
Epigenetic regulators
Inhibitor of neurogenesis
Telomere-associated protein
Ectoderm development
Mesoderm development
Endoderm development
Extra-embryonic development
Totipotency
Pluripotency
Multipotency
Unipotency
Data on KEGG graph
Rendered by Pathview

Supplemental Figure 9. Gene network analysis of overexpressed genes in GBM tissue relative to normal cortical tissue (A). Association of overexpressed genes in GBM tissue relative to normal cortical tissue in the context of maintaining pluripotency of stem cells (B).

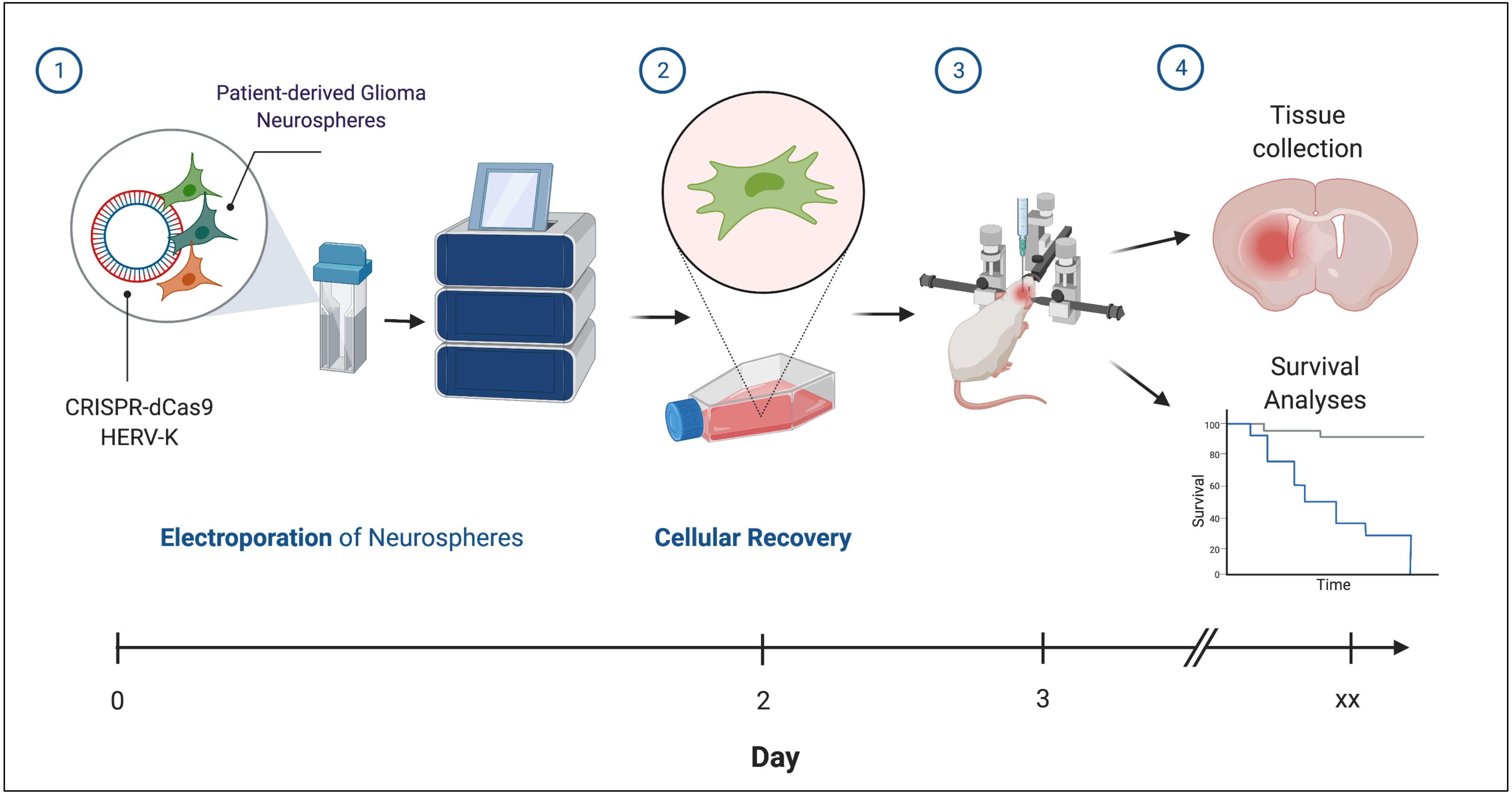
1
Patient-derived Glioma
Neurospheres
CRISPR-dCas9
HERV-K
2
3
4
Tissue
collection
Survival
Analyses
100
80
60
40
20
0
Survival
Time
Electroporation of Neurospheres
Cellular Recovery
0
2
3
xx
Day

Supplemental Figure 10. Glioma neurospheres were transfected via electroporation and allowed to recover for 3 days prior to stereotactic injection. Transfected neurospheres were implanted into the right frontal lobe of nude athymic mice (n=10 mice each group, 2E5cells/2 uL).

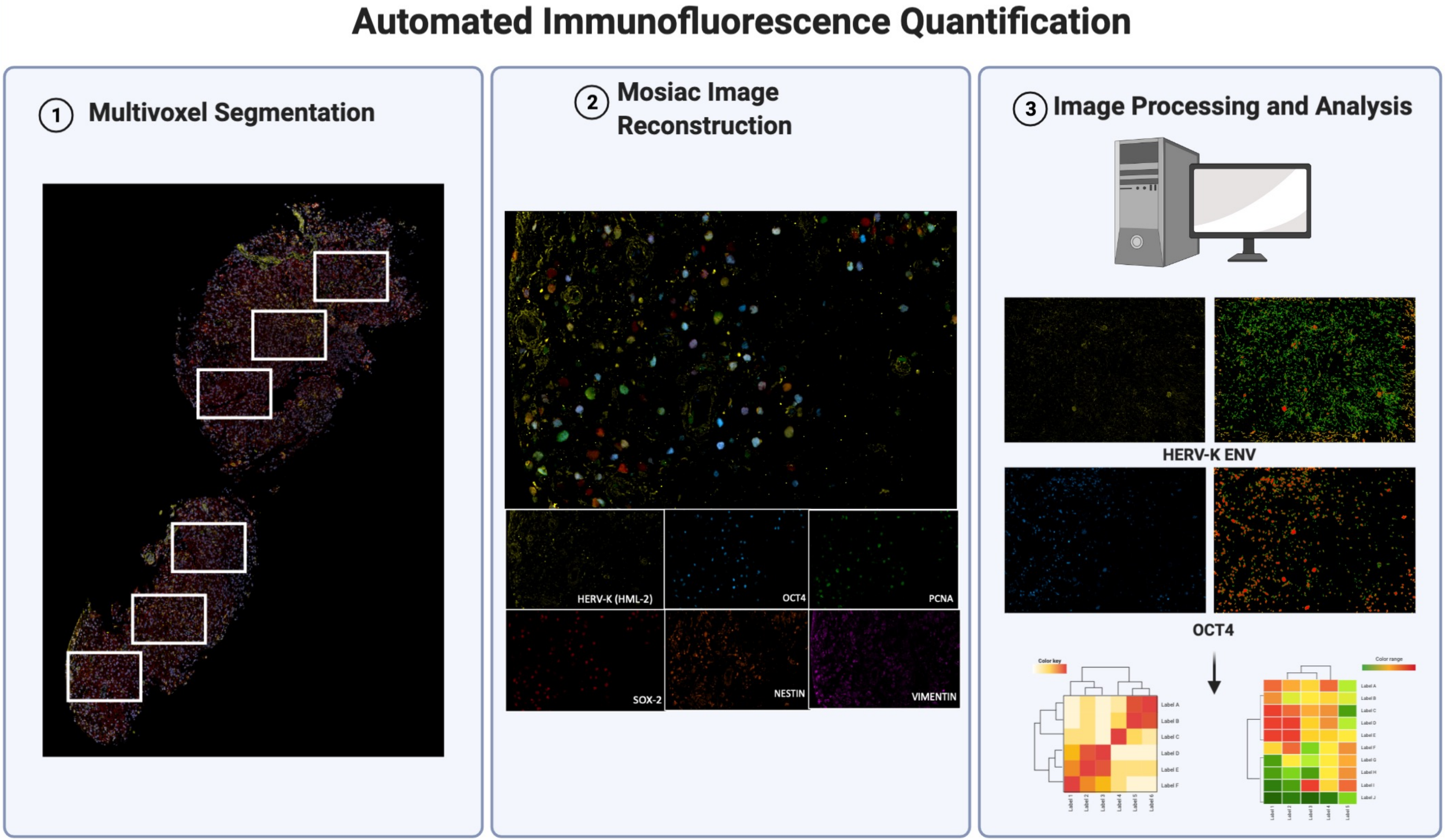

Automated Immunofluorescence Quantification
1 Multivoxel Segmentation
2 Mosiac Image Reconstruction
3 Image Processing and Analysis
HERV-K (HML-2)
OCT4
PCNA
SOX-2
NESTIN
VIMENTIN
HERV-K ENV
OCT4

Supplemental Figure 11. Automated Fluorescence Quantification Pipeline can be utilized to quantify fluorescence expression per voxel. Primarily, whole-slide multiplex immunofluorescence slides were randomly segmented and mosaic images were reconstructed for each fluorophore. Images were subsequently processed using an automated algorithm for each protein and total surface area of protein expression was quantified.

Supplemental Table 1

Patient	Age	Sex	Pathology	Recurrence	IDHm R132	Proliferative index	Tissue Sample	HERVK+ on IF
1	49	F	Diffuse Astrocytoma (WHO Grade II)	No	+	4-5%	Tumor	-
2	79	M	Glioblastoma (WHO Grade IV)	No	-	20-25%	Tumor	++
3	50	M	Glioblastoma (WHO Grade IV)	No	-	50-55%	Tumor	++
4	68	M	Glioblastoma (WHO Grade IV)	Yes	-	NP	Tumor	-
5	39	M	Glioblastoma (WHO Grade IV)	Yes	-	1-6%	Tumor	++
6	40	M	Anaplastic Astrocytoma (WHO Grade III)	No	+	10-15%	Tumor CSF	-
7	31	F	Anaplastic Astrocytoma (WHO Grade III)	Yes	+	40-50%	Tumor CSF	+
8	60	M	Glioblastoma (WHO Grade IV)	Yes	-	NP	Tumor	++
9	79	M	Glioblastoma (WHO Grade IV)	Yes	-	NP	Tumor	-
10	44	M	Glioblastoma (WHO Grade IV)	No	-	NP	Tumor	-
11	60	M	Glioblastoma (WHO Grade IV)	Yes	-	8-10%	Tumor	+
12	31	M	Glioblastoma (WHO Grade IV)	Yes	-	20%	Tumor	-
13	30	F	Anaplastic Oligodendroglioma (WHO Grade III)	No	+	15%	Tumor CSF	+
14	69	M	Glioblastoma	Yes	-	12-15%	Tumor CSF	++
15	41	M	Glioblastoma (WHO Grade IV)	Yes	+	15-30%	Tumor CSF	NA

16	54	M	Anaplastic Astrocytoma (WHO Grade III)	Yes	+	10-15%	CSF	NA
17	39	F	Anaplastic Oligodendroglioma	No	+	2%	Tumor CSF	NA
18	46	M	Diffuse Astrocytoma (Grade II)	No	-	-	Tumor CSF	NA
19	44	M	Anaplastic Oligodendroglioma	No	+	-	CSF	NA
20	50	F	Glioblastoma (WHO Grade IV)	Yes	-	-	Tumor	NA
21	39	M	Anaplastic Oligodendroglioma	Yes	+	-	Tumor	NA
22	55	M	Glioblastoma (WHO Grade IV)	Yes	-	-	Tumor	NA

Stem-like versus Differentiated			Neural Progenitor Cells (NPCs) versus Astrocytes (ACs)		
**Locus**	**log2FC**	**Adj. p-value**	**Locus**	**log2FC**	**Adj. p-value**
HML2-10q24.2	1.29	p<0.0001	HML2-1q22	1.95	p<0.0001
HML2-3q12.3	2.07	p<0.0001	HML2-1q23.3	1.44	p<0.0001
HML2-19q11	1.95	p<0.0001	HML2-11q22.1	2.11	p<0.0001
HML2-12q14.1	1.38	p<0.0001	HML2-12q14.1	2.41	p<0.0001
HML2-3q13.2	1.29	p<0.0001	HML2-19q11	2.69	p<0.0001
HML2-22q11.21	1.45	p<0.0001	HML2-22q11.21	2.21	p<0.0001
HML2-11q22.1	1.35	p<0.0001	HML2-3q13.2	2.18	p<0.0001
HML2-5p13.3	1.15	p<0.0001	HML2-3q27.2	1.62	p<0.0001
HML2-8p23.1d	1.59	p<0.0001	HML2-5p13.3	1.78	p<0.0001
HML2-3q21.2	1.20	p<0.0001	HML2-5q33.3	1.85	p<0.0001
HML2-8p23.1c	1.46	p<0.0001	HML2-6q14.1	1.65	p<0.0001
HML2-5q33.3	1.14	p<0.0001	HML2-7p22.1	1.54	p<0.0001
HML2-1q22	1.15	p<0.0001	HML2-8p23.1a	1.27	p<0.0001
			HML2-8q24.3a	1.27	p<0.0001
			HML2-3q12.3	1.47	p<0.0001
			HML2-21q21.1	1.37	p<0.0001
			HML2-10q24.2	1.27	p<0.0001
			HML2-8p23.1c	1.17	p<0.0001
			HML2-8p23.1d	1.23	p<0.0001

Supplemental Table 2. Comparison of overexpressed HML2 loci according to Johnson-Verhaak and Neftel states, respectively. In each instance, HML2 locus expression in stem-like or progenitor cells is compared to that of terminally differentiated cells.

Supplemental Table 3. Summary of HML-2 loci significantly overexpressed in NPC cells relative to mesenchymal cells (MES)

Element	Locus	log2FC	Adj. p-value
ERV-127104-LTR5-Hs	Chr1q21.3	5.75	0.0004
ERV-2578677-LTR5-Hs	Chr3q12.3	7.20	0.0007
ERV-1888790-LTR5-Hs	Chr19q13.12	6.12	0.002
ERV-3052630-HERVK-int	Chr4q35.2	6.28	0.008
ERV-1822930-LTR5-Hs	Chr19p12	3.50	0.04

Supplemental Table 4. Total HML-2 expression in NPC cells relative to those of other transcriptional states.

Transcriptional State	log2FC	Adj p-value
AC	5.54	<0.00001
MES1	7.87	<0.00001
MES2	5.08	<0.00001
OPC	5.38	<0.00001

Location of HERV-K (HML-2) active loci on scRNA-seq

HERVd Nomenclature	Locus	HML-2 Strand	Sequence Length (bp)	HML-2 Consensus Missing bp	Transcript alignment to full length HML-2 Genes
ERV-1853911	19q13	+	5,697	3,921 (45.9%)	LTR5B, RNAseH, Env, Rec
ERV-3925927	8p23.1	-	9,528	173 (1.8%)	LTR5HA, Integrase, Protease
ERV-610406	11q12.3	-	14,601	71(0.8%)	LTR5Hs, Integrase, Np9, Protease, RNAseH
ERV-3697915	7p22	-	15,438	189 (2.1%)	LTR5Hs, LTR45C, Gag, Integrase, Env, RNAseH, Rec
ERV_2925064	4q32.3	+	12,534	2628 (27.7%)	LTR5Hs, Np9
ERV-1887448	19q13.12	-	4,006	3,855 (51.2%)	Integrase, Rec
ERV_3697923	7p22.1	-	8,408	97 (1.14%)	LTR5Hs, Gag, Protease, RNAseH, Integrase
ERV_4438050	Yp11.2	-	6,944	1,629 (19.0%)	LTR5A, Integrase
ERV_3798887	8p23.1	+	9,522	92 (0.9%)	LTR5A, Integrase, Protease
ERV_3292433	5q33.2	-	8,703	917 (9.6%)	LTR5B, Protease
ERV_2289356	20q11.22	+	9,635	2,755(28.9%)	LTR5B, RNAseH
ERV_2427227	22q11.21	+	9,175	384 (4.1%)	LTR5Hs, Gag, Integrase, Np9, Protease, RNAseH
ERV_3926052	8p23.1.3	-	9,516	91 (1.0%)	LTR5A, Integrase, Rec
ERV_749060	11q22.1	+	9,466	164 (1.73%)	LTR5HS, Env, Gag, Pol, Integrase, Rec, RNAseH
ERV_1882163	19q11	-	8,864	80 (0.94%)	LTR5Hs, Env, Gag, Pol, Rec, Protease, RT

ERV_2599259	3q21.2	+	9,138	343 (3.62%)	LTR5HS, Env, Gag, Protease Rec, RNAseH

Supplemental Table 5

Supplemental Table 6

Chromosome	Start	End	Width	Strand	Gene ID	Function
Chr 1	150618701	150669672	50972	-	GOLPH3L	Golgi Trafficking
Chr 1	155629233	155755165	GOLPH3L	-	YY1AP1	Cell Cycle Regulation, Chromatin Remodeling, Transcriptional Regulation
Chr 3	125898908	125929011	30104	+	ALDH1L1-AS2	lncRNA
Chr 3	101659703	101716770	57068	+	RDUR	RIG-I Dependent Antiviral Response Regulator RNA, lncRNA
Chr 3	113005777	113160361	154585	-	CFAP44	Microtubule Cytoskeleton Organization
Chr 4	68566996	68946669	379674	+	UBA6-DT	Ubiquitination and Proteasomal Degradation
Chr 5	136310987	136835018	524032	-	SPOCK1	Proteoglycan Protein Core
Chr 8	139142266	139509065	366800	-	FAM135B	Cellular Lipid Metabolism
Chr 8	12394588	12523120	128533	-	LOC729732	lncRNA
Chr 9	136627016	12523120	230431	-	VAV2	Guanine Nucleotide Exchange Factor
Chr 11	62369691	62380237	10547	-	EML3	Microtubule Binding Activity
Chr 11	62382768	62389647	6880	-	B3GAT3	Glucuronyltransferase
Chr 11	62360675	62369312	8638	-	MTA2	Nucleosome Remodeling
Chr 12	58325232	58329947	4716	-	GIHCG	lncRNA, Associated with Hepatocellular Carcinoma
Chr 19	35549963	35597208	47246	-	HPN-AS1	lncRNA
Chr 19	20278023	20311299	33277	+	ZNF486	DNA-binding Transcription Factor Activity
Chr 20	34129778	34145405	15628	+	ERGIC3	Golgi to ER Vesicle Transport
Chr 22	23522552	23660224	137673	+	BCR	Serine-Threonine Kinase Activity and GTPase-activating Protein

Supplemental Table 7

HML2 Consensus vs. Sham Vector – CD34+ Astroglia: Significantly Upregulated Pathways

**Pathways**	**Enrichment P Value**	**Activation Predicted By Z-Score**
ILK Signaling	0.0045	ACTC1, DSP, IRS4, KRT18, LEF1, MYL1
Transcriptional Regulatory Network in Embryonic Stem Cells	0.0081	CDX2, H4C14, H4C15
Ethanol Degradation IV	0.015	ALDH1A2, GPX7
Dopamine Degradation	0.025	ALDH1A2, SULT1A3/SULT1A4
NAD Signaling Pathway	0.028	H2BC12,H2BC15,H2BU1,POLR2A
Phenylethylamine Degradation I	0.030	AOC2
Immunogenic Cell Death Signaling Pathway	0.032	HSPA1A/HSPA1B,HSPA6,PYCARD
BMP Signaling Pathway	0.032	BMP3,HOXC9,SOSTDC1
VEGF Signaling	0.040	ACTC1,FLT1,PTPN6
NER (Nucleotide Excision Repair, Enhanced Pathway)	0.046	H4C14,H4C15,POLR2A
Tight Junction Signaling	0.048	ACTC1,CGN,MYL1,OCLN

Supplemental Table 8

HML2 Consensus vs. Sham Vector – CD34+ Astroglia: Significantly Upregulated Functions

**Pathways**	**Enrichment P Value**	**Z-Score**
Tumorigenesis of Epithelial Neoplasm	0.000392	0.205
Transcription of DNA	0.0000694	1.365
Proliferation of neuronal cells	0.00162	0.392
Organismal Death	0.000000613	-1.656
Neoplasia of tumor cell lines	0.00359	1.293
Neoplasia of cells	0.000257	1.354
Necrosis	0.00000285	-0.498
Migration of Cells	0.00000237	1.102
Invasion of tumor cell lines	0.00365	1.575
Invasion of Cells	0.0000417	1.417
Incidence of Tumor	0.000125	0.335
Growth of Epithelial Tissue	0.000081	0.902
Generation of Tumor	0.000393	0.191
Expression of RNA	0.00076	1.716
Differentiation of Stem Cells	0.00223	-0.481

Development of Malignant Tumor	0.000125	0.281
Cell Viability	0.000838	2.432
Cell Transformation	0.000131	1.092
Cell Survival	0.000964	2.513
Cell Proliferation of Tumor Cell Lines	0.00278	0.391
Cell Movement	0.00000459	1.235
Cancer of Cells	0.00401	1.884
Apoptosis of Tumor Cell Lines	0.000134	-0.161
Advanced Malignant Tumor	0.00000071	-0.065
Activation of DNA Endogenous Promoter	0.000063	1.042

Supplemental Table 9

CRISPR-dCas9-HERV-K	Sequence
Sequence 1	GATAGGGAAAAACCGCCTTAAGGG
Sequence 2	AAAGCAGTATTGCTGCCCGCAGG
Sequence 3	TCCTGCCTGTCCCTGGGCAATGG
Sequence 4	AGTAGATGGAGCATACAATCGGG

Supplemental Table 10

Primary Antibodies

Target	Host	Use	Manufacturer/ Product #	Dilution
HERV-K Env	Mouse	WB, IF	Austral Biologics HERM 1855	1:500 (WB) 1:500 (IF)
Nestin	Mouse	WB, IF	Millipore Sigma MAB5326	1:1000 (WB) 1:100 (IF)
OCT4 (POUF1A)	Rabbit	WB, IF	Millipore Sigma ABD116	1:1000(WB) IF(1:500) 1:1000(ChIP)
Sox-2	Rat	IF	ThermoFisher 14-9811-82	1:100 (IF)
PCNA	Mouse	IF	GeneTex GTX40237	1:100 (IF)
Vimentin	Chicken	IF	Millipore Sigma AB5773	1:100 (IF)
S-100b	Rabbit	IF	Novus Biologics NBP1-87102	1:100 (IF)
GFAP	Mouse	IF	BD Biosciences 556330	1:1000 (IF)
GAPDH	Mouse	WB	Abcam ab8245	1:2000 (WB)

Supplemental Table 11

Target	Primers 5'-3'
HML-2 ENV	F: CTGCCAAACCTGAGGAAGAA<br>R: ACCAACCAATTTTGGACTGC
HML-2 POL	F: TCACATGGAAACAGGCAAAA<br>R: AGGTACATGCGTGACATCCA
HML-2 LTR5HS	F: GTTTGTCTGCTGACCCTCTC<br>R: AGCCTCTGAGTTCCCTTAGT
OCT4 (POU5F1)	F: GGTTCTCGATACTGGTTCGC<br>R: GTGGAGGAAGCTGACAACAA
Nestin	F: CAGCGTTGGAACAGAGGTTGG<br>R: TGGCACAGGTGTCTCAAGGGTAG
B-Actin	F: ATCGAGCACGGCATCGTCA<br>R: AGCACAGCCTGGATAGCAAC

Supplemental Table 12

Target	Locus
HML-2 ENV PRIMER BINDING SITES (hg38)	chr16:34231555+34231672<br>chr19:22762054+22762171<br>chr22:18932768+18932885<br>chr1:160667209+160667326<br>chr1:75846546+75846663<br>chr3:125616011+125616128<br>chr3:101417265+101417382<br>chr10:101581695-101581812<br>chr12:58723722-58723839<br>chr19:36064850-36064967<br>chr19:28131005-28131122<br>chr21:19935424-19935541<br>chr1:207810924-207811041<br>chr1:155598937-155599054<br>chr3:112745597-112745714<br>chr3:9891839-9891956<br>chr5:156087197-156087314<br>chr5:30489232-30489349<br>chr6:78429134-78429248<br>chr7:4633041-4633158<br>chr7:4624537-4624654<br>chr8:7357877-7357994

Supplemental Table 13

RNA-Scope Multiplex v2 Probes

Target	Channel	Color	Dilution
HERV-K Env	C1	Green	1:1500
Nestin	C2	Amber	1:1500
OCT4 (POUF1A)	C3	Red	1:1500

Supplemental Table 14

Sample	Figure	Statistical Test	
HERV-K ENV RNA CSF	1I	Un-paired t-test	35.2±8.8 vs. 23.1±6.7, n=18, p=0.02, technical triplicates
HERV-K ENV RNA-tumor tissue	IJ	Mann-Whitney Test	1.15±0.2 vs. 0.5±0.2, p=0.01, n=16, technical triplicates
HERV-K ENV qPCR-cell lines	1K	One-way ANOVA	ANOVA, p<0.03, technical and biological triplicates
Summary expression of all HERV-K(HML-2) ORF loci	2E-F	One-way ANOVA	p<0.001, Bonferroni Multiple-testing correction,
CRISPR-d-Cas9-HERV-K qPCR	5B	One-way ANOVA	*p<0.05, **p<0.01, ***p<0.001, ****p<0.0001, technical and biological triplicates, multiple testing correction
Intracranial Patient-derived Xenograft Model	6J	Log-rank (Kaplan-Meier)	OS: 26 days vs. 18.6, p=0.0008, n=10 per group
Reverse Transcriptase in Extracellular Vesicles	7C	One-way ANOVA	p<0.001, biological and technical triplicates
Abacavir effects on Reverse Transcriptase	7D	One-way ANOVA	*p<0.05, **p<0.01, biological and technical triplicates
HML2 consensus vs. sham vector – CD34+ astroglia HERV-K ENV	6F	Mann-Whitney Test	29.85±16.9 vs. 1.00±16.9, p<0.03, four replicates
HML2 consensus vs. sham vector – CD34+ astroglia OCT4	6F	Mann-Whitney Test	1.86±0.2 vs. 1.00±0.2, p<0.03, four replicates

Supplemental Methods

Unbiased Clustering of HML-2 ORF expression

All 72 HML-2 loci containing open reading frames (ORFs), along with stem cell signatures were included in an unbiased analysis of potential HML-2 markers. The Seurat function FindMarkers was used to identify differentially expressed HML-2 loci in OPC/AC and OPC clusters relative to MES1, MES2, and AC/MES1 clusters. Subsequently, HML-2 loci differentially expressed in all three comparison clusters (MES1, MES2, AC/MES1) were mapped using the Seurat function FeaturePlot.

Digital Droplet PCR

The digital PCR reaction was set in 96-well plates in duplicate in an AutoDG Droplet Digital PCR System (Bio-Rad) with a set of primers and probe (FAM labeled) to detect HERV-K env (forward primer: 5' ATTTGGTGCCAGGAACTGAG 3'; reverse primer: 5' GCTGTCTCTTCGGAGCTGTT 3' and probe 5' 6-FAM-AGGAGTTGCTGATGGCCTCG Iowa Black FQ 3'). For the analysis of CSF samples, to confirm the extracellular origin of HML-2 DNA in CSF, a pre-made assay of primers and probes targeting a cellular DNA (RPP30 gene, HEX-tagged) was also included (Bio-Rad, 10031244). For the analysis of HML-2 RNA in brain tissue and cell lines, HPRT1 was used as a reference gene (HEX-tagged, Bio-Rad pre-made assay, Catalogue number: 10031256). The master mix was composed of 12.5 µl of ddPCR Supermix (no dUTP) (Bio-Rad), 1.25 µl of a mix of HERV-K env primers (900 nm) and probe (250 nm) (Bio-Rad), 1.25 µl of RPP30 or HPRT1 assay (Bio-Rad), 2.5 µl of cDNA and 7.5 µl of RNAse-free water. After preparing the droplets, the PCR was performed in a T100 Thermal cycler (Bio-Rad) with the following cycling conditions: 95°C for 10 minutes, 40 cycles of 95°C for 30 seconds and 60°C for 1 minute, and 95°C for 10 minutes. The number of copies was determined in a QX200 Digital PCR reader (Bio-Rad).

Chromatin Immunoprecipitation (ChIP)

Prior to ChIP, GBM 28 and 43 were cultured according to methods above. Fifteen million cells were split and washed with PBS, centrifuged and resuspended in 10mL of fresh serum-free media. Cells were fixed by adding 18.5% paraformaldehyde to a final concentration of 1% for 10 minutes at room temperature. Cells were quenched using 1.25M of glycine and rocked at room temperature for 5 minutes. Cells were washed twice with DPBS, resuspended in the 1mL ChIP lysis buffer and

incubated on ice for 10 minutes. To isolate nuclei, cells were spun at 5000 RPM for 5 minutes at 4°C. Nuclei were then resuspended in 200 µL of the nuclear lysis buffer and incubated for 10 minutes on ice. To shear chromatin into ~500bp fragments, cells were sonicated for 10 seconds pulses (3x) at 30% power with 30 seconds rest on ice. Chromatin was centrifuged at 14,000 RPM for 10 minutes at 4°C, and supernatant was collected and diluted 1:5 in ChIP dilution buffer. 100 µL of this was transferred to another tube and stored in -80°C as input. Magnetic beads (Dynabeads, Invitrogen) were pre-washed (3x), and immunoprecipitated using 5 µg of non-specific IgG, RNA polymerase II, and anti-OCT-4. After overnight incubation, the beads were washed with a low salt buffer followed by a high salt buffer and Tris-HCL/EDTA buffer (3x for 3 minutes each). Elution buffer was added to samples and inputs and incubated for 30 minutes at 65°C shaking at 1000 RPM. To separate protein/DNA complex, proteinase K (20 mg/mL) was added for 2 hrs with shaking at 55°C. Phenol-chloroform extraction was performed. To precipitate DNA, 2µL of glycogen was added and samples were stored in -80°C overnight. Pellets were washed with ethanol and resuspended in nucleic acid-free H_2O. Samples were quantified using RT-qPCR.

Supplemental Video 1

HML-2 induces significant changes in cellular morphology and satellitosis. Images taken over 72 hours in 3D culture.
